# A computational examination of the therapeutic advantages of fourth-generation ALK inhibitors TPX-0131 and repotrectinib over third-generation lorlatinib for NSCLC with ALK F1174C/L/V mutations

**DOI:** 10.3389/fmolb.2023.1306046

**Published:** 2024-01-11

**Authors:** Ambritha Balasundaram, George Priya C. Doss

**Affiliations:** Laboratory of Integrative Genomics, Department of Integrative Biology, School of BioSciences and Technology, Vellore Institute of Technology, Vellore, Tamil Nadu, India

**Keywords:** anaplastic lymphoma kinase, non-small-cell lung cancer, mutations, F1174C/L/V, lorlatinib, TPX-0131, repotrectinib

## Abstract

**Background:** In non-small-cell lung cancer (NSCLC), a pivotal factor in promoting cancer development is the rearrangement in the anaplastic lymphoma kinase *ALK* gene, resulting in elevated ALK protein expression. F1174C/L/V is the acquired secondary resistant mutation in ALK. Significant survival improvements have been seen while tyrosine kinase inhibitors specifically target ALK. Nevertheless, the emergence of drug resistance hinders the clinical effectiveness of these drugs.

**Objective:** This research sought to find the binding affinity/inhibitory effects of the existing drug lorlatinib (LOR) and upcoming TPX-0131 (zotizalkib/TPX) and repotrectinib (TPX-0005/REP) inhibitors against ALK F1174C/L/V mutations using computational approaches to identify potential strategies over resistance.

**Methods:** We conducted molecular docking, molecular dynamics simulation, and MMPBSA calculations to investigate how compact macrocyclic inhibitors, such as TPX-0131 and repotrectinib, fit within the ATP-binding boundary and differ from LOR.

**Results:** Our results demonstrated that TPX-0131 and repotrectinib contributed to higher binding energy in F1174C and F1174L mutations than LOR. Repotrectinib showed greater binding energy in the F1174V mutation, whereas LOR and TPX-0131 exhibited similar binding energy. However, all three inhibitors showed significant binding energy toward F1174C/L/V mutations found in NSCLC.

**Conclusion:** This comparative study of the potential binding effects of fourth-generation inhibitors TPX-0131 and repotrectinib and third-generation inhibitor LOR for ALK F1174C/L/V mutations revealed the atomistic insights of the binding mechanism. These computational findings enable us to carry out further research for the clinical implementation of fourth-generation ALK inhibitors on ALK-positive NSCLC.

## 1 Introduction

Non-small-cell lung cancer (NSCLC) has a distinct molecular subtype defined by anaplastic lymphoma kinase (ALK) rearrangements, found in around 3%–7% of NSCLC cases. The most frequently observed change in the *ALK* gene involves its fusion with echinoderm microtubule-associated protein-like 4 (EML4) (Soda et al., 2007). Furthermore, more than 20 fusion genes have been identified and documented, including TGF-ALK, KIF5B-ALK, and STRN-ALK. ALK-positive NSCLC has been linked to factors such as lack of smoking history, younger age, and adenocarcinoma histology (Shaw et al., 2009; Pan et al., 2021). As of January 2020, the literature had revealed a comprehensive list of 90 distinct ALK fusion partners, spanning both coding and noncoding RNAs in NSCLC (Ou et al., 2020). ALK inhibitors have recently become more effective and selective, changing the treatment paradigm for advanced ALK-positive NSCLC, leading to prolonged patient survival. There are three generations of the six ALK tyrosine kinase inhibitors (TKIs). The US Food and Drug Administration (FDA) and European Medicines Agency (EMA) have approved the use of these drugs in advanced ALK-positive NSCLC treatment. These ALK TKIs include crizotinib (first generation), ceritinib, brigatinib, alectinib, and ensartinib (second generation), and lorlatinib (third generation) (Remon et al., 2022; Rijavec et al., 2022).

Lorlatinib belongs to the third generation of oral TKIs. It operates through reversible and ATP-competitive mechanisms, targeting ALK and ROS1. Unlike the second-generation inhibitors, lorlatinib was deliberately engineered to penetrate the central nervous system (CNS) and overcome existing secondary resistance mutations within the ALK tyrosine kinase domain. In preclinical investigations, lorlatinib has exhibited greater potency than earlier-generation TKIs when dealing with non-mutated ALK. Furthermore, it maintains its efficacy against well-known individual ALK resistance mutations, such as the highly challenging G1202R solvent front mutation (Johnson et al., 2014; Zou et al., 2015; Shaw et al., 2019). Even though next-generation (lorlatinib) ALK TKIs have higher kinase selectivity and an improved capacity to overcome drug resistance, therapeutic resistance is proven to unavoidably develop after a specific period following the beginning of medication administration. Research indicates that around 35% of the acquired resistance to lorlatinib mechanisms is attributed to compound mutations (Zou et al., 2015; Shaw et al., 2017a; Solomon et al., 2018). Recent studies revealed that 6 out of 14 compound mutations exhibited sensitivity to first- or second-generation ALK TKI inhibitors. However, approximately fifty percent of the compound mutations resisted every ALK TKI medication currently on the market. Notable examples of such resistant mutations include combinations such as G1202R + F1174C, G1202R + F1174L, and G1202R + L1196M, among others (Yoda et al., 2018; Okada et al., 2019; Song et al., 2021). Turning Point Therapeutics, Inc. recently developed TPX-0131 (zotizalkib) and repotrectinib (TPX-0005), two tiny and compact macrocyclic compounds. These two compounds have a lower molecular weight than currently available FDA-approved ALK TKIs. These two compounds are classified as fourth-generation ALK TKIs due to their high potency against various mutations resistant to lorlatinib (Song et al., 2021).

The predominant ALK mutation, F1174C/L (16.7%), has been previously identified in ALK-positive NSCLC (Katayama et al., 2012; Friboulet et al., 2014). F1174 mutations in ALK are situated near the C-terminus of the αC helix and may enhance an active conformation, thereby elevating the ATP-binding affinity of ALK. A study uncovered a novel secondary acquired mutation, ALK F1174V, through comprehensive next-generation sequencing in an ALK-positive NSCLC patient who exhibited disease progression on crizotinib administration following an extended partial response (Ou et al., 2014). The ALK kinase domain hotspot mutation F1174 (mutated to C, I, L, S, or V) is found in around 85% of cases involving ALK mutations in neuroblastoma. Developed as second-generation ALK inhibitors, ceritinib and alectinib were specifically designed to address resistance issues associated with initial ALK inhibitors. However, resistant mutations to these drugs have been observed, such as C1156Y/T, F1174C/L/V, I115ITins L1152P/R, I1171T/N/S, and G1202R for ceritinib in NSCLC and I1171T/N/S and G1202R for alectinib. The L1196M gatekeeper mutation remains the most common resistance to crizotinib (Li et al., 2018). The ALK F1174C/L/V mutation is a secondary acquired resistance to crizotinib in lung cancer (Arbour and Riely, 2017; Kim et al., 2017). Crizotinib, a first-generation ALK TKI, is the primary therapy for ALK rearrangement in NSCLC (Solomon et al., 2014). A study has reported that several ALK mutations, including E1129V, F1174C, F1174L, F1174V, I1171T, and G1269A, can emerge as resistance mechanisms during treatment with crizotinib but showed positive response to brigatinib. Brigatinib use was halted after 10 months when the patient’s overall health deteriorated, coinciding with the identification of EML4-ALK variant 1 and ALK F1174C through liquid biopsy. However, it was observed that the ALK F1174C mutation conferred resistance to brigatinib. Notably, ALK F1174C has also been reported to be sensitive to lorlatinib and alectinib. A phase II study involving advanced ALK-positive NSCLC patients with various F1174 missense mutations (F1174C/I/L/M/S/V, etc.) responded well to lorlatinib (Hu et al., 2022).

There has not been any prior computational study that has delved into the precise implications of F1174C/L/V mutations on the binding process of fourth-generation drugs TPX-0131 and repotrectinib against the third-generation drug lorlatinib. Therefore, the current research uses molecular docking and molecular dynamics simulation (MDS) techniques to elucidate the mechanisms underlying the impact of third- and fourth-generation drug binding on ALK F1174C/L/V mutations.

## 2 Materials and methods

### 2.1 Molecular docking

Molecular docking was conducted to better understand the different binding modes and quantify the binding affinities in terms of binding free energies of third-generation and fourth-generation drugs at the binding site of ALK WT and F1174C/L/V mutations (Morris et al., 2008). We obtained the three-dimensional structure of ALK protein PDB ID: 4FOB with a resolution of 1.90 Å from the RCSB Protein Data Bank (Lewis et al., 2012; Rose et al., 2013). The co-crystallized water molecules and the bounded ligand were then removed from the structure using PyMOL software (Lill and Danielson, 2011). Using Swiss PDB Viewer, we further mutated the structure to F1174C/L/V, and energy minimization of each structure was carried out using the GROMOS-96 force field (Kaplan and Littlejohn, 2001). The 3D structures of lorlatinib, TPX-0131, and repotrectinib are obtained in the SDF format from PubChem (Kim et al., 2021). Using AutoDock 4.2.6, a molecular docking experiment was carried out with a grid box of active sites centered on the ATP-binding residues of the ALK protein (Morris et al., 2009). During docking, AutoDock software initially assigned specific hydrogens, charges, and flexible torsions to the proteins and ligands. Polar H-atoms were introduced to the target proteins to precise amino acid ionization and tautomeric states. Kollam and Gasteiger charges were assigned to proteins and ligands, respectively. In addition to assigning rigid roots to the ligand, five bonds were rendered rotatable. The modified 3D proteins and ligands allowed for the flexibility of its bonds, which were saved in the PDBQT format as necessary in AutoDock4 and AutoDock Vina for docking calculations (Trott and Olson, 2010). AutoGrid4.2 generated a grid box, and the grid information was saved in grid.txt format. AutoDock Vina utilized ligand and protein information and grid box properties in the setup file. Both ligands and proteins are considered as rigid throughout the docking phase. After the docking procedure was complete, 10 configuration files containing the 10 optimal docking postures for protein–ligand were acquired for each. The drugs with the minimum binding energy (kcal/mol) and the smallest root-mean-square deviation (RMSD) were selected as the best docking poses. The Discovery Studio was used to visualize protein–ligand interactions (Biovia, 2017). Additionally, the inhibition constant (Ki) was determined based on the binding energy (ΔG) using the following formula: Ki (µM) = exp (ΔG/RT), where R is the gas constant (1.985 × 10^−3^ kcal mol^-1^ K^−1^) and T is the temperature (298.15 K) (Ali et al., 2023).

### 2.2 Molecular dynamics simulation

MDS was performed for the ALK WT and F1174C/L/V mutations to get insights into the stability of these mutations using GROMACS 2018 (Van Der Spoel et al., 2005). Furthermore, the best docking poses from the molecular docking findings were subjected to MDS to gain structural insights into the stability of the ALK F1174C/L/V mutations with third- and fourth-generation drugs**.** We generated 4 APO and 12 complex systems for MDS: i) WT–APO, ii) WT–LOR, iii) WT–TPX, iv) WT–REP, v) F1174C–APO, vi) F1174C–LOR, vii) F1174C–TPX, (viii) F1174C–REP, ix) F1174L–APO, x) F1174L–LOR, xi) F1174L–TPX, xii) F1174L–REP, (xiii) F1174V–APO, xiv) F1174V–LOR, xv) F1174V–TPX, and xvi) F1174V–REP. The CHARMM-GUI solution builder generated the input files for MDS via CHARMM force field features for protein (Jo et al., 2008). The ligand topology was generated through the ParamChem server using the CHARMM General Force Field (CGenFF) (Vanommeslaeghe et al., 2010). Initially, we uploaded the 3D coordinates of the ligand structures (inhibitors) in PDB/MOL2 format. Subsequently, the parameterization process occurred, involving the assignment of partial charges and force field parameters. Following this, the topology and coordinate files for the inhibitors were generated. We then incorporated these generated ligand topology and coordinate files into CHARMM input files for MDS. Five phases are included in the CHARMM-GUI solution builder. The tool initially reads the coordinates of the protein–ligand complex. In the second phase, the protein–ligand complex is solvated in a cubic box of TIP3P waters. To neutralize the systems, Na^+^ and Cl^−^ ions are introduced. The third phase establishes the periodic boundary conditions (PBCs), which are used to approximate a larger system by utilizing a unicell that is duplicated in all directions. Short minimization is used in this phase to eliminate bad contacts. Long-range electrostatic interactions were managed using the particle mesh Ewald (PME) approach, and non-bounded interactions were handled utilizing a distance of 12 Å cut-off and buffered using the Verlet cut-off scheme (Essmann et al., 1995). Before the simulation, the system’s energy consumption was reduced to a minimum by using the steepest descent technique (5,000 steps). In the fourth phase, the equilibration takes place in two steps, namely, the NVT ensemble and the NPT ensemble, to confirm that the system has reached the precise temperature and pressure (125 ps at 303.15 k). The temperature and pressure are sustained by the Nose–Hoover thermostat and the Parrinello–Rahman barostat (Parrinello and Rahman, 1981). The system is subjected to an NPT ensemble for 100 ns at 303.15 k and 1 bar. The final phase is the MD production phase, which comprises the number of steps of the MD run. The 16 systems generated from the CHARMM-GUI were used as input files and performed in GROMACS 2018 for a 200 ns MD production run using the CHARMM27 force field.

#### 2.2.1 Trajectory analysis

Following the MDS, tools from the GROMACS package were used to study the obtained trajectories. The gmx rms, gmx rmsf, gmx gyrate, gmx sasa, and gmx H-bond tools were used to study the RMSD, root-mean-square fluctuation (RMSF), radius of gyration (Rg), solvent-accessible surface area (SASA), and Hydrogen bond (H-bond) formation in the ALK WT and F1174C/L/V APOs and complexes. Using the gmx do_dssp function, the change in the protein structures between the ALK WT and F1174C/L/V mutations, along with different ligand-bound states, was also examined.

#### 2.2.2 Principal component analysis and Gibbs free energy surface

Principal component analysis (PCA) is a widely used analytical technique for examining dimensionality reduction in large datasets (David and Jacobs, 2014). This method is used to depict the functional motions of biomolecules. The covariance matrix eigenvalues and eigenvectors underwent diagonalization and were solvated to provide the principal components (PCs) for all the systems. The eigenvalues and eigenvectors showed the motion’s magnitude and directions. The gmx covar was used to determine the covariance matrix, which was constructed and diagonalized using the gmx covar function. The gmx anaeig function was used to determine the overlap between the estimated principal components and the trajectory’s coordinates. The Gibbs free energy surface (FES) was used to detect changes in the protein’s possible conformations and Gibbs free energy for the ALK WT and F1174C/L/V APOs and complexes. The FES was calculated using the gmx sham function and the probability distribution of the first two eigenvectors (Iida et al., 2016). Furthermore, a Python script displayed the outcomes in 3D graphics.

#### 2.2.3 Dynamic cross-correlation matrix and molecular mechanics/Poisson–Boltzmann surface area calculation

The dynamic cross-correlation matrix (DCCM) signifies the correlation coefficient’s magnitude, which depends on how closely the system’s fluctuations are connected. The protein structure’s MD trajectory was used to compute the residue cross-covariance matrix for the atomic fluctuations. Strong diagonal relativity, spreading from the diagonal, and off-diagonal cross-relationships are some of the main properties of the DCCM. The correlated motions of residues were examined to assess the quality of protein structures.

The molecular mechanics/Poisson–Boltzmann surface area (MMPBSA) calculation method is a standard technique for determining the binding free energy of a protein–ligand complex. The stable region of the last 50 ns MD trajectory was utilized to compute the components of the binding energy via the g_mmpbsa tool (Kumari et al., 2014). Free energy related to protein–ligand complex binding can be expressed as

$$\Delta G_{\text{binding}}=G_{\text{complex}}\;–\;\left(G_{\text{protein}}+G_{\text{ligand}}\right).$$

Here, G_complex_ stands for the complex’s overall binding energy, and G_protein_ and G_ligand_ stand for the complex’s unbound protein and ligand energy, respectively. Moreover, we assessed the energy contribution of amino acid residues to the interaction. In MMPBSA calculations, the res.dat file serves as a repository of data detailing the contributions of individual amino acid residues to the overall free energy of a molecular interaction, such as protein–ligand binding. Using these res.dat values, we generated graphs where each residue in a protein is plotted on the *x*-axis against its corresponding energy contribution on the *y*-axis. These binding energy calculations for individual residues were conducted on the simulated complexes to identify the amino acid residues crucial for ligand binding. Generally, a more negative value signifies a stronger molecule interaction, indicating a favorable and energetically stable binding. Conversely, a positive value suggests an unfavorable or weaker interaction, signaling that the binding is less stable or less energetically favorable.

## 3 Results

### 3.1 Molecular docking and binding energy analysis

**FIGURE 1:**
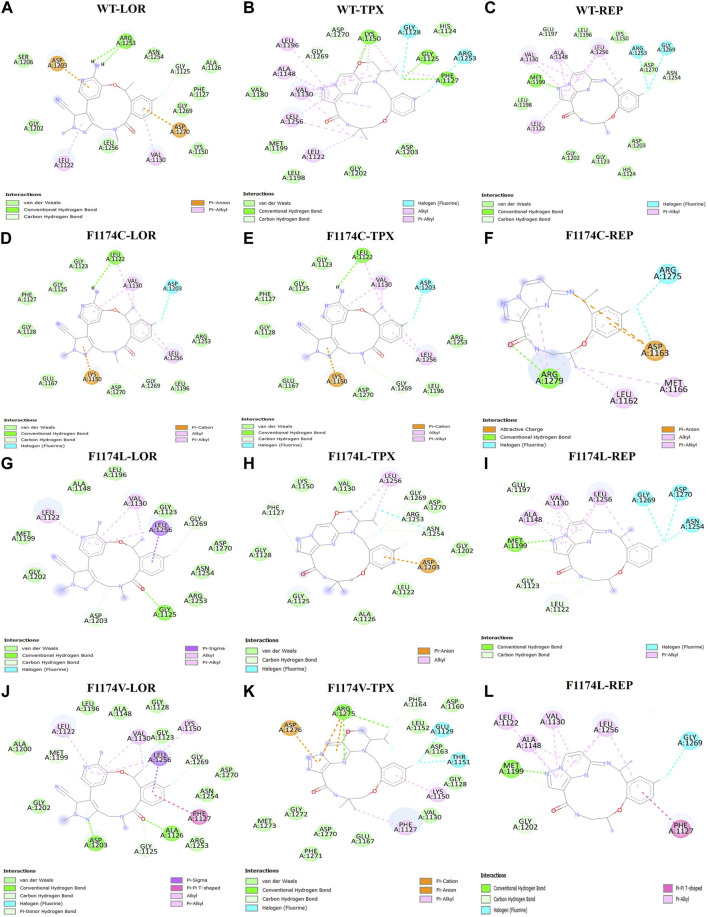
2D structural representation of the molecular interaction of ALK WT with **(A)** LOR, **(B)** TPX, and **(C)** REP. The 2D structural representation of the molecular interaction of ALK F1174C with **(D)** LOR, **(E)** TPX, and **(F)** REP. The 2D structural representation of the molecular interaction of ALK F1174L with **(G)** LOR, **(H)** TPX, and **(I)** REP. The 2D structural representation of the molecular interaction of ALK F1174V with **(J)** LOR, **(K)** TPX, and **(L)** REP.

The molecular docking software AutoDock4 and AutoDock Vina were used to evaluate the binding affinity and comparative inhibitory properties of the WT and ALK F1174C/L/V mutations with the selected third- and fourth-generation drugs. The obtained binding energy, constant of inhibition (Ki), and the H-bond interactions of the selected conformations are presented in Table 1. A lower binding energy indicates a better and more stable drug–receptor interaction. The inhibitor dissociation constant (Ki) is an equilibrium constant of an irreversible inhibitor for interaction with its target protein. The lower the Ki value in the reaction equilibrium between the receptor and the drug, the more the action equilibrium favors the formation of receptor–compound complexes. According to the findings of molecular docking, the WT-REP complex had the highest binding energy of −9.8 kcal/mol, followed by WT–TPX (−9.5 kcal/mol) and WT–LOR (−8.9 kcal/mol). In the F1174C mutation, the F1174C–LOR complex had the highest binding energy of −8.6 kcal/mol, followed by F1174C–TPX (−7.7 kcal/mol) and F1174C–REP (−6.9 kcal/mol). In the F1174L mutation, the F1174C–REP complex had the highest binding energy of −9.7 kcal/mol, followed by F1174L–LOR (−8.4 kcal/mol) and F1174L–TPX (−7.8 kcal/mol). In the F1174V mutation, the F1174V–REP complex had the highest binding energy of −10.1 kcal/mol, followed by F1174L–LOR (−7.3 kcal/mol) and F1174L–TPX (−7.7 kcal/mol). We observed lower Ki values in F1174C–LOR, F1174C–REP, F1174L–TPX, F1174V–LOR, F1174V–TPX, and F1174L–REP complexes compared to others. Furthermore, we examined the complex structures in 2D interaction using Discovery Studio, and the protein–drug interactions are shown in Figure 1 and Table 1.

**TABLE 1:** Molecular docking result analysis of ALK WT and F1174C/L/V mutations complexed with LOR, TPX, and REP inhibitors. Comparative binding energy (Kcal/mol), Ki values, and H-bond interactions between the ALK F1174C/L/V mutations and LOR, TPX, and REP inhibitors.

	Binding energy (kcal/mol)	Ki values	Hydrogen bond		
			Amino acid residues	H-bond monitoring	Distance (Å)
WT–LOR	−8.9	299.41 nM	ARG1253	LOR:H7—A:ARG1253:O and LOR:H8—A:ARG1253:O	2.71 and 2.74
WT–TPX	−9.5	108.76 nM	GLY1125, PHE1127, and LYS1150	A:GLY1125:HN—TPX:F2, A:PHE1127:HN—TPX:F2, and A:LYS1150:HZ2—TPX:O4	2.12, 2.62, and 1.92
WT–REP	−9.8	65.55 nM	MET1199	A:MET1199:H—REP:N8	1.93
F1174C–LOR	−8.6	2.27 uM	LEU1122	LOR:H—A:LEU1122:O	2.9
F1174C–TPX	−7.7	496.78 nM	LEU1122	A:GLY1269:HN—TPX:F3	2.68
F1174C–REP	−6.9	8.76 uM	ARG1279	A:ARG1279:HE—REP:O3	2.66
F1174L–LOR	−8.4	696.25 nM	GLY1125	A:GLY1125:HN—:LOR:O2	2.58
F1174L–TPX	−7.8	1.92 uM	—	—	—
F1174L–REP	−9.7	77.60 nM	MET1199	A:MET1199:H—REP:N8	2.36
F1174V–LOR	−7.3	4.46 uM	ALA1126 and ASP1203	A:ALA1126:HN—LOR:O2	1.85
F1174V–TPX	−7.7	2.27 uM	ARG1275	A:ARG1275:HH11—TPX:F1 and A:ARG1275:HH22—TPX:N10	2.30 and 2.83
F1174V–REP	−10.1	39.51 nM	MET1199	A:MET1199:H—REP:N8	2.55

### 3.2 MDS for ALK WT and F1174C/L/V mutation APOs and complexes

MDS was carried out for ALK WT–APO and F1174C/L/V mutations to determine the structural instability caused by these substitutions in ALK. Furthermore, MDS was conducted to explore the LOR, TPX, and REP drugs’ binding affinities and conformational changes for the ALK WT and F1174C/L/V mutations. A total of 16 MDS runs were conducted for 200 ns, including 4 APOs and 12 complexes, and the RMSD, RMSF, Rg, SASA, and intramolecular H-bond were initially assessed (Table 2).

**TABLE 2:** Average values of RMSD, RMSF, Rg, SASA, and the number of intramolecular H-bonds over 200 ns MDS for ALK WT and F1174C/L/V mutations with and without inhibitors.

	RMSD (nm)	RMSF (nm)	Rg (nm)	SASA (nm^2^)	Number of intramolecular H-bonds
WT–APO	0.24 ± 0.04	0.14 ± 0.08	2.09 ± 0.02	163.9 ± 3.08	194 ± 8
WT–LOR	0.27 ± 0.03	0.12 ± 0.07	2.08 ± 0.02	156.84 ± 3.43	211 ± 8
WT–TPX	0.27 ± 0.04	0.15 ± 0.09	2.09 ± 0.02	158.99 ± 3.21	195 ± 8
WT–REP	0.18 ± 0.02	0.10 ± 0.06	2.05 ± 0.01	158.14 ± 2.86	218 ± 7
F1174C–APO	0.28 ± 0.03	0.13 ± 0.08	2.10 ± 0.01	164.6 ± 2.93	194 ± 7
F1174C–LOR	0.36 ± 0.05	0.18 ± 0.12	2.08 ± 0.02	164.08 ± 3.22	192 ± 7
F1174C–TPX	0.28 ± 0.05	0.16 ± 0.11	2.09 ± 0.01	159.38 ± 3.86	199 ± 7
F1174C–REP	0.27 ± 0.05	0.15 ± 0.08	2.10 ± 0.02	160.34 ± 3.26	196 ± 8
F1174L–APO	0.23 ± 0.03	0.14 ± 0.08	2.05 ± 0.01	160.72 ± 3.32	194 ± 7
F1174L–LOR	0.33 ± 0.06	0.15 ± 0.08	2.11 ± 0.02	164.44 ± 2.99	198 ± 8
F1174L–TPX	0.23 ± 0.04	0.12 ± 0.07	2.09 ± 0.02	159.86 ± 3.33	201 ± 7
F1174L–REP	0.29 ± 0.04	0.15 ± 0.10	2.10 ± 0.02	158.50 ± 3.84	197 ± 7
F1174V–APO	0.23 ± 0.04	0.14 ± 0.06	2.09 ± 0.02	159.93 ± 3.11	199 ± 8
F1174V–LOR	0.30 ± 0.10	0.18 ± 0.12	2.06 ± 0.02	160.66 ± 3.61	196 ± 8
F1174V–TPX	0.29 ± 0.05	0.13 ± 0.08	2.06 ± 0.01	155.51 ± 3.23	198 ± 7
F1174V–REP	0.24 ± 0.06	0.15 ± 0.08	2.08 ± 0.02	161.93 ± 3.47	199 ± 8

#### 3.2.1 Stability and flexibility analysis of inhibitor binding versus APO in ALK F1174C/L/V

**FIGURE 2:**
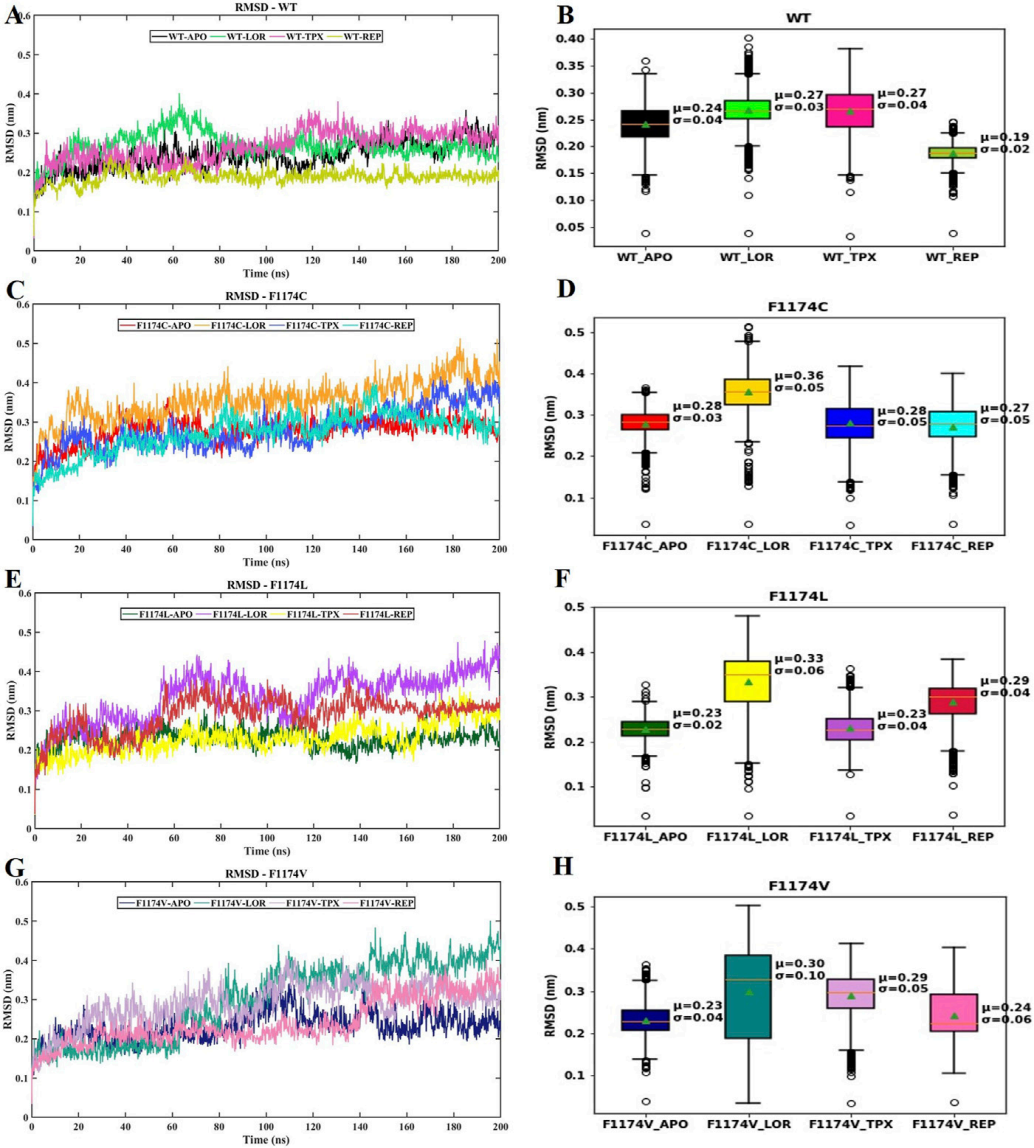
RMSD of protein backbone atoms for ALK WT–APO and F1174C/L/V mutations with and without ligands. **(A)** Comparison of the RMSD of backbone atoms for WT–APO, WT–LOR, WT–TPX, and WT–REP **(B)** The box plots depict the RMSD analysis of WT–APO, WT–LOR, WT–TPX, and WT–REP. **(C)** Comparison of the RMSD of backbone atoms for F1174C–APO, F1174C–LOR, F1174C–TPX, and F1174C–REP. **(D)** The box plots depict the RMSD analysis of F1174C–APO, F1174C–LOR, F1174C–TPX, and F1174C–REP. **(E)** Comparison of the RMSD of backbone atoms for F1174L–APO, F1174L–LOR, F1174L–TPX, and F1174L–REP. **(F)** The box plots depict the RMSD analysis of F1174L–APO, F1174L–LOR, F1174L–TPX, and F1174L–REP. **(G)** Comparison of the RMSD of backbone atoms for F1174V–APO, F1174V–LOR, F1174V–TPX, and F1174V–REP. **(H)** The box plots depict the RMSD analysis of F1174V–APO, F1174V–LOR, F1174V–TPX, and F1174V–REP. In the box plots, the whisker bars indicate the minimum and maximum RMSD ranges, the median is shown by a line splitting the box, and the mean (μ) with standard deviation (σ) values are mentioned inside the plot.

The protein backbone’s RMSD was computed over the simulation period to ensure structural stability. The RMSD curve and box plot with mean, median, and standard deviation for all the structures are shown in Figure 2. The RMSD mean of all the structures did not exceed 3.6 nm, showing that the complexes were stable during the simulation period (Table 2). The RMSD curve flattened after 60 ns in all the simulations, which signifies that all the structures have reached equilibrium, and we can proceed with further analysis (Figure 2; Table 2).

**FIGURE 3:**
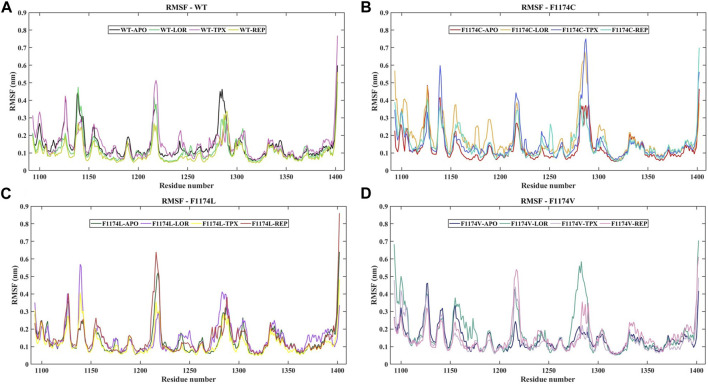
RMSF of cα-atoms for ALK WT and F1174C/L/V mutations with and without inhibitors. **(A)** The RMSF of cα-atoms for WT–APO, WT–LOR, WT–TPX, and WT–REP. **(B)** The RMSF of cα-atoms for F1174C–APO, F1174C–LOR, F1174C–TPX, and F1174C–REP. **(C)** The RMSF of cα-atoms for F1174L–APO, F1174L–LOR, F1174L–TPX, and F1174L–REP. **(D)** The RMSF of cα-atoms for F1174V–APO, F1174V–LOR, F1174V–TPX, and F1174V–REP. The *x*-axis represents the number of residues, while the *y*-axis indicates the RMSF in nanometers (nm).

**FIGURE 4:**
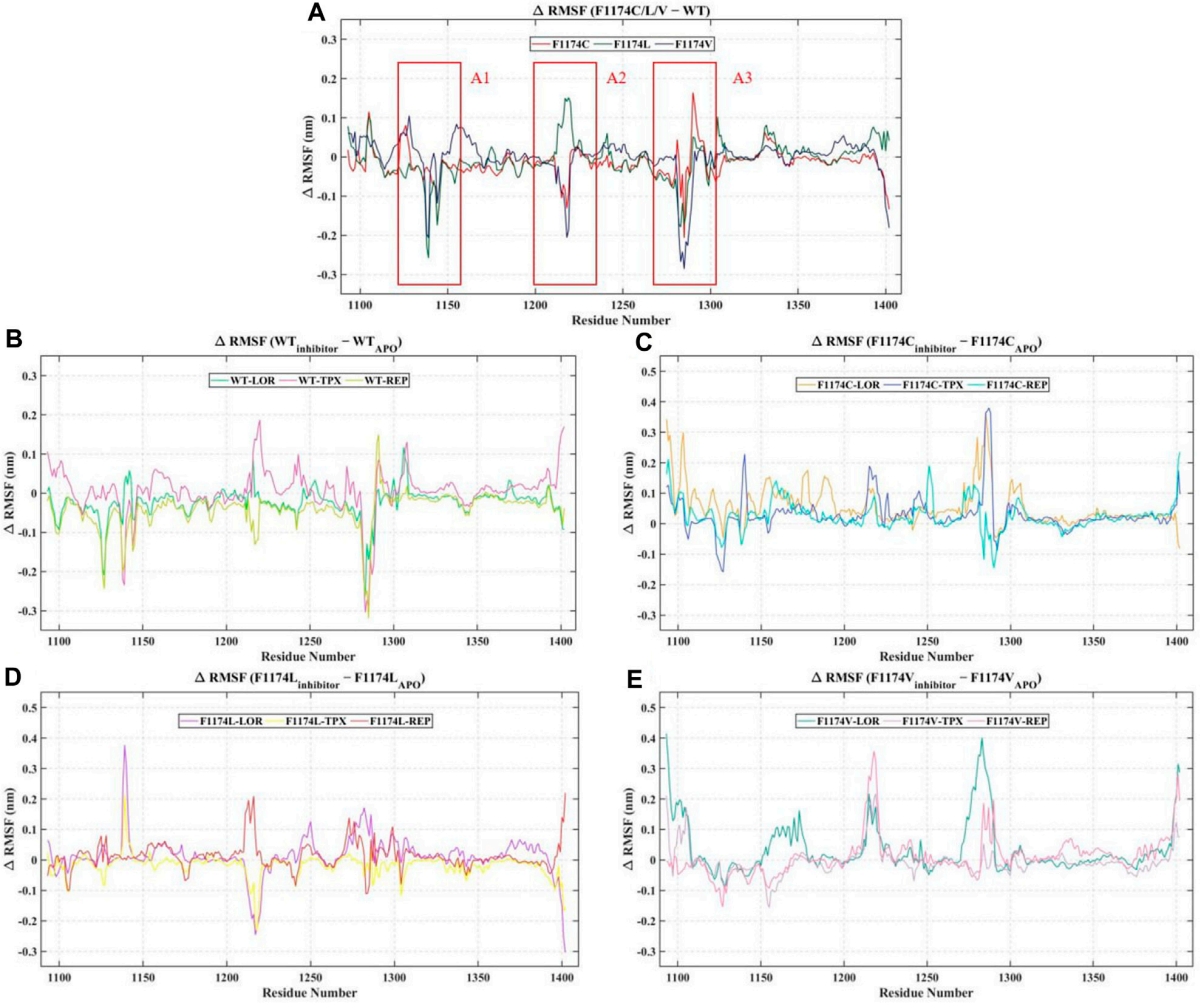
Analysis of change in the per residue RMSF. **(A)** The differences in the RMSF (Δ RMSF) of residues of the ALK WT–APO against the F1174C/L/V mutations **(B)** The Δ RMSF of the ALK WT–APO residues against the ALK WT complex with inhibitors LOR, TPX, and REP. **(C)** The Δ RMSF of the ALK F1174C–APO residues against the ALK F1174C complex with inhibitors LOR, TPX, and REP. **(D)** The Δ RMSF of the ALK F1174L–APO residues against the ALK F1174L complex with inhibitors LOR, TPX, and REP. **(E)** The Δ RMSF of the ALK F1174V–APO residues against the ALK F1174V complex with inhibitors LOR, TPX, and REP.

The protein Cα atoms’ RMSF values were measured for all APOs to analyze the flexibility of the protein residues. The WT–APO projected a mean RMSF of 0.14 ± 0.08, while F1174C, F1174C, and F1174C mutations projected a mean RMSF of 0.13 ± 0.08, 0.14 ± 0.08, and 0.14 ± 0.06, respectively (Figure 3; Table 2). For comparison, we determined the Δ RMSF (RMSF_F1174C/L/V-APO_—RMSF_WT-APO_) of the F1174C/L/V mutations concerning WT–APO, as shown in Figure 4A. The regions with more negative values suggest increased rigidness in the mutations, whereas those with the most positive values indicate flexibility. We found rigidness in the A1 region of F1174L/V, the A2 region of F1174C/V, and the A3 region of F1174C/L/V. In contrast, F1174L showed flexibility around the A2 region. Furthermore, the RMSF was measured for all 12 complexes using Cα atoms to investigate an inhibitor’s binding-mediated effects on ALK WT and F1174C/L/V structural flexibility against the APO state (Figure 3). The WT–APO projected a mean RMSF of 0.14 ± 0.08, while WT–LOR, WT–TPX, and WT–REP projected a mean RMSF of 0.12 ± 0.07, 0.15 ± 0.09, and 0.10 ± 0.06, respectively (Figure 3A). The TPX inhibitor-bounded complex exhibited higher flexibility, while the LOR and REP inhibitors exhibited rigidity in WT. The F1174C–APO projected a mean RMSF of 0.13 ± 0.08, while F1174C–LOR, F1174C–TPX, and F1174C–REP projected a mean RMSF of 0.18 ± 0.12, 0.16 ± 0.11, and 0.15 ± 0.08, respectively (Figure 3B). All the inhibitor-bounded complexes exhibited rigidity in F1174C. The F1174L–APO projected a mean RMSF of 0.14 ± 0.08, while F1174L–LOR, F1174L–TPX, and F1174L–REP projected a mean RMSF of 0.15 ± 0.08, 0.12 ± 0.07, and 0.15 ± 0.10, respectively (Figure 3C). The TPX inhibitor-bounded complex showed higher flexibility here, while LOR and REP exhibited rigid structures in F1174L. F1174V–APO projected a mean RMSF of 0.14 ± 0.06, while F1174V–LOR, F1174V–TPX, and F1174V–REP projected a mean RMSF of 0.18 ± 0.12, 0.13 ± 0.08, and 0.15 ± 0.08, respectively (Figure 3D). The TPX inhibitor-bounded complex showed higher flexibility, while the LOR and REP complexes exhibited rigidity in F1174V. For comparison, we determined the Δ RMSF (RMSF_WT-inhibitors_-RMSF_WT-APO_, RMSF_F1174C-inhibitors_-RMSF_F1174C-APO_, RMSF_F1174L-inhibitors_-RMSF_F1174L-APO_, and RMSF_F1174V-inhibitors_-RMSF_F1174V-APO_) of the F1174C/L/V complexes concerning its corresponding APOs, as shown in Figures 4B–D.

#### 3.2.2 Compactness analysis

**FIGURE 5:**
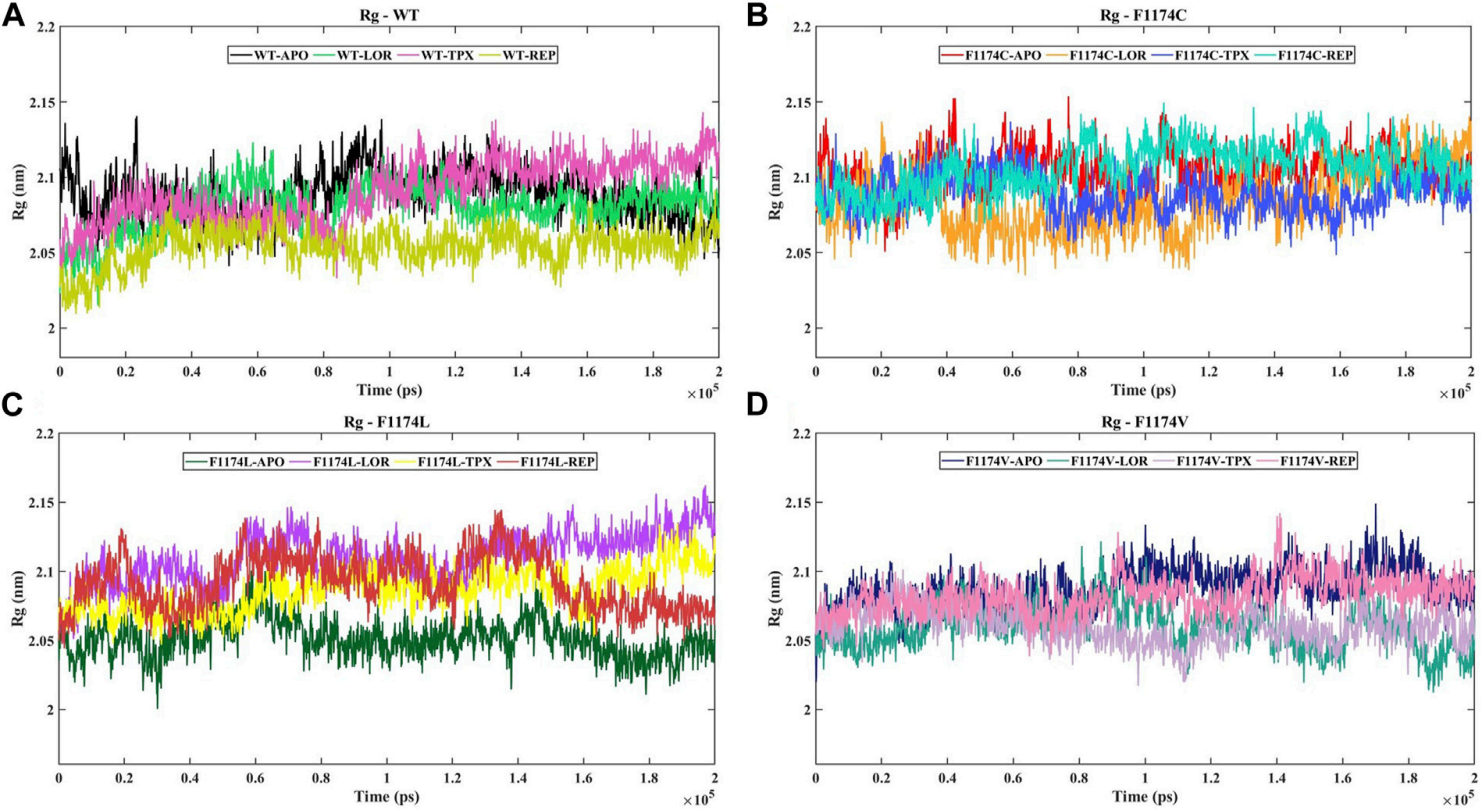
Time evolution of the Rg for ALK WT and F1174C/L/V mutations with and without inhibitors. **(A)** The Rg comparison of WT–APO, WT–LOR, WT–TPX, and WT–REP. **(B)** The Rg comparison of F1174C–APO, F1174C–LOR, F1174C–TPX, and F1174C–REP. **(C)** The Rg comparison of F1174L–APO, F1174L–LOR, F1174L–TPX, and F1174L–REP. **(D)** The Rg comparison of F1174V–APO, F1174V–LOR, F1174V–TPX, and F1174V–REP. The *x*-axis represents the time in ps, while the *y*-axis indicates the Rg in nanometers (nm).

The Rg is linked to a protein’s 3D structure and overall shape, helping us understand how compactly the protein is folded and its folding properties. We calculated the Rg for ALK WT and F1174C/L/V APOs and complexes to examine the stability of the structures. A larger Rg shows a more extended protein structure, whereas a lower Rg shows a more compact protein structure. The Rg mean for WT–APO, F1174C–APO, F1174L–APO, and F1174V–APO was calculated as 2.09 ± 0.02, 2.10 ± 0.01, 2.05 ± 0.01, and 2.09 ± 0.02, respectively. The protein structure is tightly packed in WT–APO, F1174C–APO, and F1174V–APO but has a slightly stretched structure in F1174C–APO (Table 2). The graphs of Rg data between the APOs and the complexes are shown in Figure 5. WT–REP had a slightly stretched structure compared to WT–APO, while WT–LOR and WT–TPX did not exhibit significant changes. In F1174C, there are no significant differences in the complexes compared to F1174C–APO. In F1174L, the structure is tightly packed in all the complexes compared to F1174L–APO. In F1174V, the structures are slightly stretched compared to F1174V–APO (Figure 5; Table 2).

#### 3.2.3 Solvent-accessible surface area analysis

**FIGURE 6:**
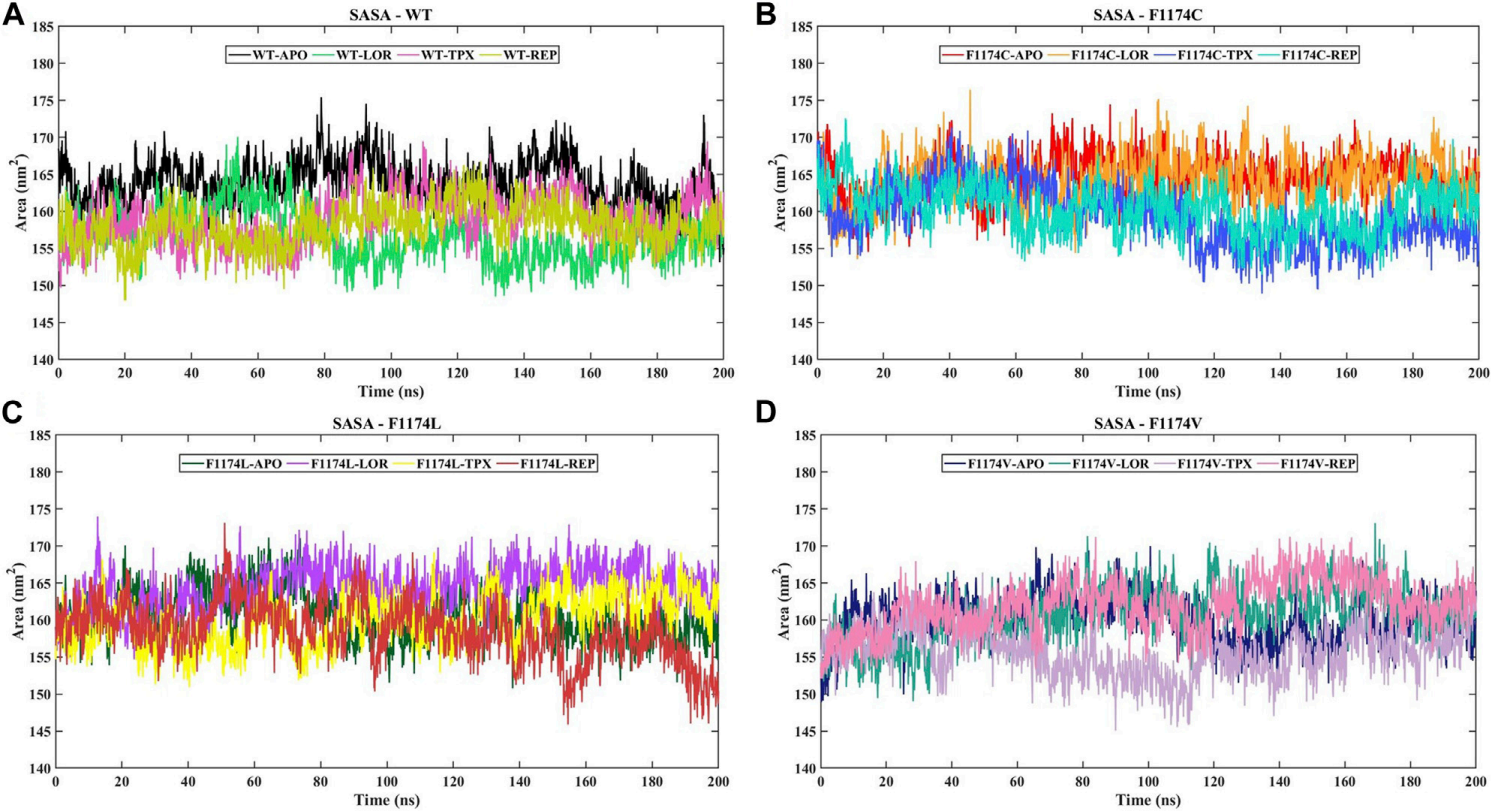
SASA of ALK WT and F1174C/L/V mutations with and without inhibitors. **(A)** The SASA comparison of WT–APO, WT–LOR, WT–TPX, and WT–REP. **(B)** The SASA comparison of F1174C–APO, F1174C–LOR, F1174C–TPX, and F1174C–REP. **(C)** The SASA comparison of F1174L–APO, F1174L–LOR, F1174L–TPX, and F1174L–REP. **(D)** The SASA comparison of F1174V–APO, F1174V–LOR, F1174V–TPX, and F1174V–REP. The *x*-axis represents the time in nanoseconds (ns), while the *y*-axis indicates the SASA in square nanometers (nm^2^).

The SASA acts as the interface between the protein and the solvent it is in contact with because of its electrostatic and surface properties. A protein’s conformational dynamics under solvent circumstances can be studied using the solvent on the system surface. The solvent property can vary depending on the situation (drug binding or mutations). A higher SASA value indicates an expansion of the structure, and the solvent accessibility increases in the surface area of the protein. We calculated the SASA for ALK WT and F1174C/L/V APOs and complexes to examine their conformational dynamics during the simulations (Figure 6; Table 2). The SASA mean for WT–APO, F1174C–APO, F1174L–APO, and F1174V–APO was calculated as 163.9 ± 3.08 nm^2^, 164.6 ± 2.93 nm^2^, 160.72 ± 3.32 nm^2^, and 159.93 ± 3.11 nm^2^, respectively (Table 2). F1174C mutation caused a higher solvent surface area, whereas F1174L and F1174V mutations caused the least solvent surface area. A remarkable rise in the mean SASA of the mutant complexes compared to the WT complexes indicates that the most surface area is accessible to solvent in the mutant complexes compared to those in the WT, with the exclusion of F1174L–REP and F1174V–REP.

#### 3.2.4 Dynamics of intramolecular and intermolecular H-bond analyses

The intramolecular H-bonds within the protein are vital in defining the stability of the protein’s 3D structure. The intramolecular H-bonds were calculated for ALK WT and F1174C/L/V APOs and complexes to understand the stability of the protein upon mutations and inhibitor binding (Supplementary Figure S1). The mean intramolecular H-bonds for WT–APO, F1174C–APO, F1174L–APO, and F1174V–APO are 194 ± 8, 194 ± 7, 194 ± 7, and 199 ± 8, respectively (Table 2). There are no significant changes in the number of intramolecular H-bonds upon F1174C/L/V mutations. The mean intramolecular H-bonds for WT inhibitor-bounded complexes WT–LOR, WT–REP, and WT–APO are 211 ± 8, 195 ± 8, and 218 ± 7, respectively (Table 2). We observed increased intramolecular H-bonds in the complexes, indicating that the binding of inhibitors makes the structure more stable. The mean numbers of intramolecular H-bonds for F1174C inhibitor-bounded complexes F1174C–LOR, F1174C–TPX, and F1174C–REP are 192 ± 7, 199 ± 7, and 196 ± 8, respectively (Table 2). Thus, in LOR binding to F1174C, we noticed a slight disruption in the number of H-bonds. The mean intramolecular H-bonds for F1174L–LOR, F1174L–TPX, and F1174L–REP are 198 ± 8, 201 ± 7, and 197 ± 7, respectively (Table 2). In the case of F1174L, the binding of inhibitors makes the structure more stable. The mean intramolecular H-bonds for F1174V inhibitor-bounded complexes F1174V–LOR, F1174V–TPX, and F1174V–REP are 196 ± 8, 198 ± 7, and 199 ± 8, respectively (Table 2). TPX/REP binding to F1174C makes the structure more stable than LOR.

**FIGURE 7:**
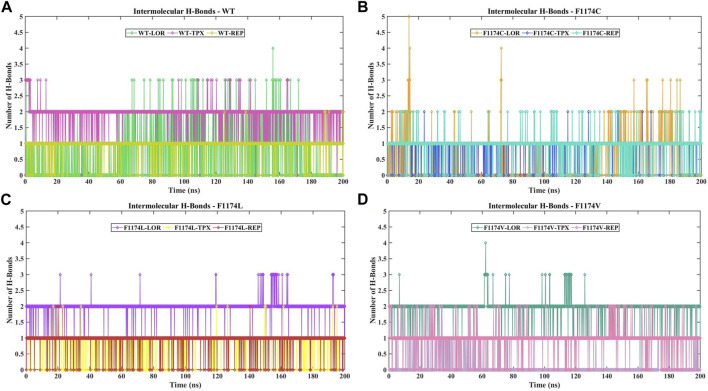
Analysis of the number of intermolecular H-bond formations in ALK WT and F1174C/L/V mutations complexed with inhibitors. **(A)** The comparison of the number of intermolecular H-bond formations in WT–LOR, WT–TPX, and WT–REP. **(B)** The comparison of the number of intermolecular H-bond formations in F1174C–LOR, F1174C–TPX, and F1174C–REP. **(C)** The comparison of the number of **i**ntermolecular H-bond formations in F1174L–LOR, F1174L–TPX, and F1174L–REP. **(D)** The comparison of the number of intermolecular H-bond formations in F1174V–LOR, F1174V–TPX, and F1174V–REP. The *x*-axis represents the time in nanoseconds (ns), while the *y*-axis indicates the number of intermolecular H-bonds formed.

The protein–ligand interactions were examined throughout the simulation using intermolecular H-bonds, which are crucial in protein–ligand binding and are intricate in investigating the stability of the protein–ligand complex to assess molecular recognition, directionality, and interaction specificity. The maximum number of intermolecular H-bonds formed for WT–LOR, WT–TPX, WT–REP, F1174C–LOR, F1174C–TPX, F1174C–REP, F1174L–LOR, F1174L–TPX, F1174L–REP, F1174V–LOR, F1174V–TPX, and F1174V–REP is 4, 3, 2, 5, 2, 2, 3, 2, 2, 4, 2, and 2, respectively (Figure 7). During the simulation, intermolecular H-bonds persist significantly in WT–TPX, WT–REP, F1174C–TPX, F1174C–REP, F1174L–LOR, and F1174V–LOR.

#### 3.2.5 Essential dynamics analysis

**FIGURE 8:**
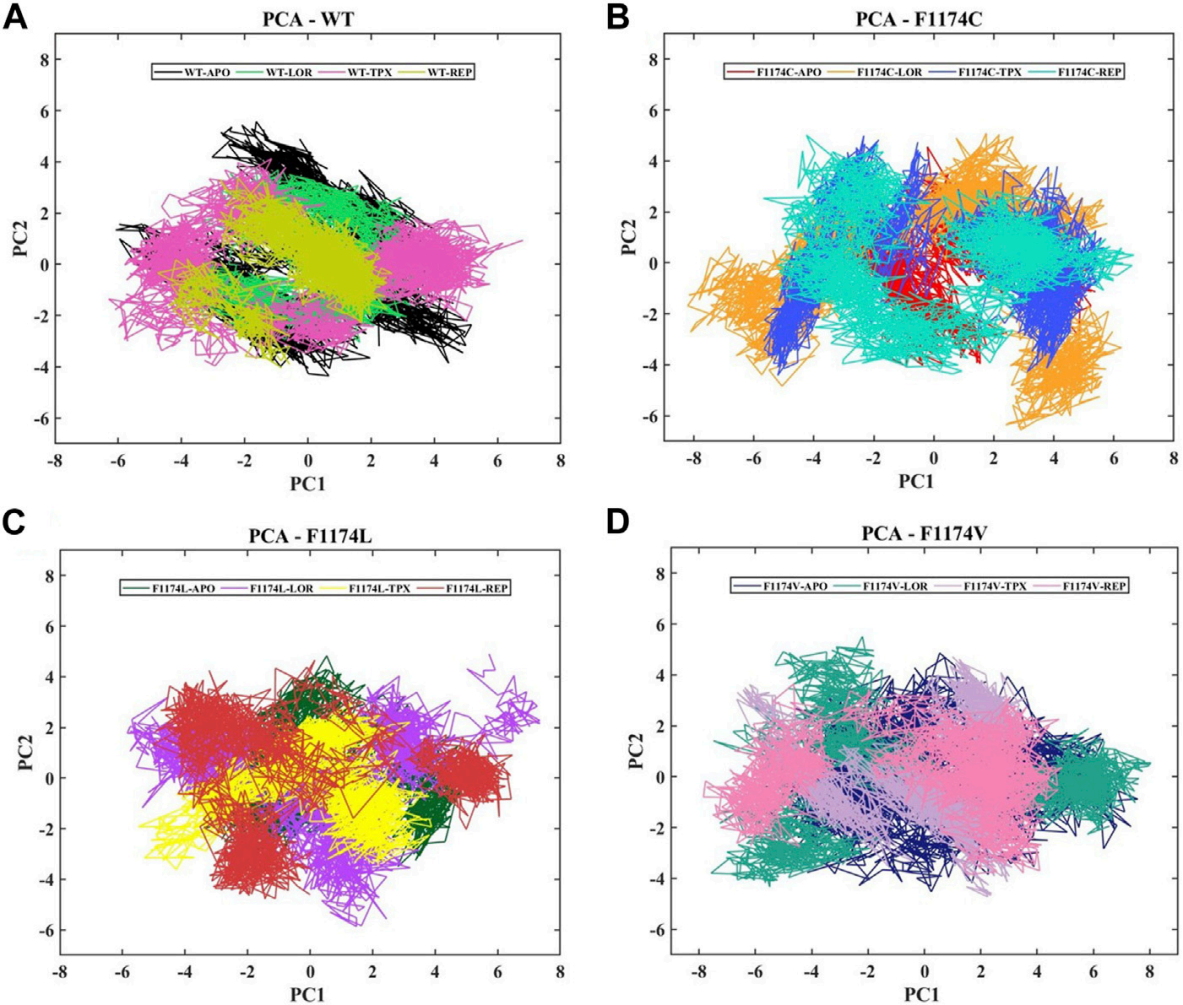
PCA was conducted on the ALK WT and the F1174C/L/V mutations, both in the presence and absence of inhibitors. **(A)** The PCA comparison of WT–APO, WT–LOR, WT–TPX, and WT–REP. **(B)** The PCA comparison of F1174C–APO, F1174C–LOR, F1174C–TPX, and F1174C–REP. **(C)** The PCA comparison of F1174L–APO, F1174L–LOR, F1174L–TPX, and F1174L–REP. **(D)** The PCA comparison of F1174V–APO, F1174V–LOR, F1174V–TPX, and F1174V–REP. The *x*-axis represents the first principal component (PC1) in nanometers (nm), while the *y*-axis indicates the second principal component (PC2) in nanometers (nm).

A protein’s fundamental dynamics are governed by switching between distinct configurations, and the phenomena driving this modularity of the protein are governed by overall collective movements. The covariance matrix of the eigenvectors is diagonalized in this dimensionality reduction approach to establish a projection of the two PCs, PC1 and PC2, which describe the subspace where most protein dynamics occur. The collective motion of the ALK WT and F1174C/L/V APOs and complexes in the MD trajectories was investigated using PCA to assess the structural and conformational changes brought by inhibitor binding. Figure 8 shows the dynamic protein movements calculated by PCA. Compared to WT–APO, F1174C/L mutations in the APO state occupied a reduced subspace; this decreased arbitrary motion of the proteins leads to compact structures. In contrast, F1174V does not show major deviations in the subspace, but motion variations were noticed compared to WT. The PCA results suggest that the underlying cause for protein function impairment may decrease/change the overall motion in F1174C/L/V mutations because proteins execute their functions through coordinated atomic movements, and a protein’s stability is connected to its collective atomic motion. In the case of WT–APO, the PCs lie between 5.17 and −6.08 on PC1 and 5.57 to −4.35 on PC2, while in its WT–LOR, WT–TPX, and WT–REP, the motion ranges from 4.32 to −4.63 on PC1 and 3.78 to −3.05 on PC2, 6.80 to −6.30 on PC1 and 4.12 to −3.96 on PC2, and 2.66 to −4.75 on PC1 and 3.37 to −4.04 on PC2, respectively. The WT–TPX occupied a larger phase space and flexibility than other complexes. For F1174C–APO, the PCs lie between 5.38 and −3.17 on PC1 and 4.55 and −4.08 on PC2, while in its F1174C–LOR, F1174C–TPX, and F1174C–REP, the motion ranges from 6.25 to −8.20 on PC1 and 5.08 to −6.55 on PC2, 5.69 to −5.66 on PC1 and 4.99 to −4.41 on PC2, and 6.67 to −5.68 on PC1 and 5.01 to −4.35 on PC2, respectively. The F1174C mutation caused slightly reduced subspace; all the bound inhibitors occupied a larger phase space and showed more flexibility than F1174C–APO. For F1174L–APO, the PCs lie between 5.25 and −3.41 on PC1 and 4.82 and −3.50 on PC2, while in its F1174L–LOR, F1174L–TPX, and F1174L–REP, the motion ranges from 7.35 to −5.84 on PC1 and 4.90 to −5.86 on PC2, 3.88 to −5.49 on PC1 and 3.01 to −3.61 on PC2, and 6.47 to −5.02 on PC1 and 4.65 to −4.77 on PC2, respectively. The F1174L mutation showed a reduced subspace compared to WT–APO, and all the bound inhibitor complexes showed more flexibility than F1174L–APO. For F1174V–APO, the PCs lie between 7.57 and −5.85 on PC1 and 4.85 and −4.52 on PC2, while in its F1174V–LOR, F1174V–TPX, and F1174V–REP, the motion ranges from 8.03 to −7.57 on PC1 and 5.51 to −4.34 on PC2, 3.71 to −6.66 on PC1 and 4.71 to −4.72 on PC2, and 4.89 to −7.84 on PC1 and 3.64 to −3.84 on PC2, respectively. The F1174V complexes showed increased phase and flexibility compared to F1174V–APO.

#### 3.2.6 Gibbs free energy surface analysis

**FIGURE 9:**
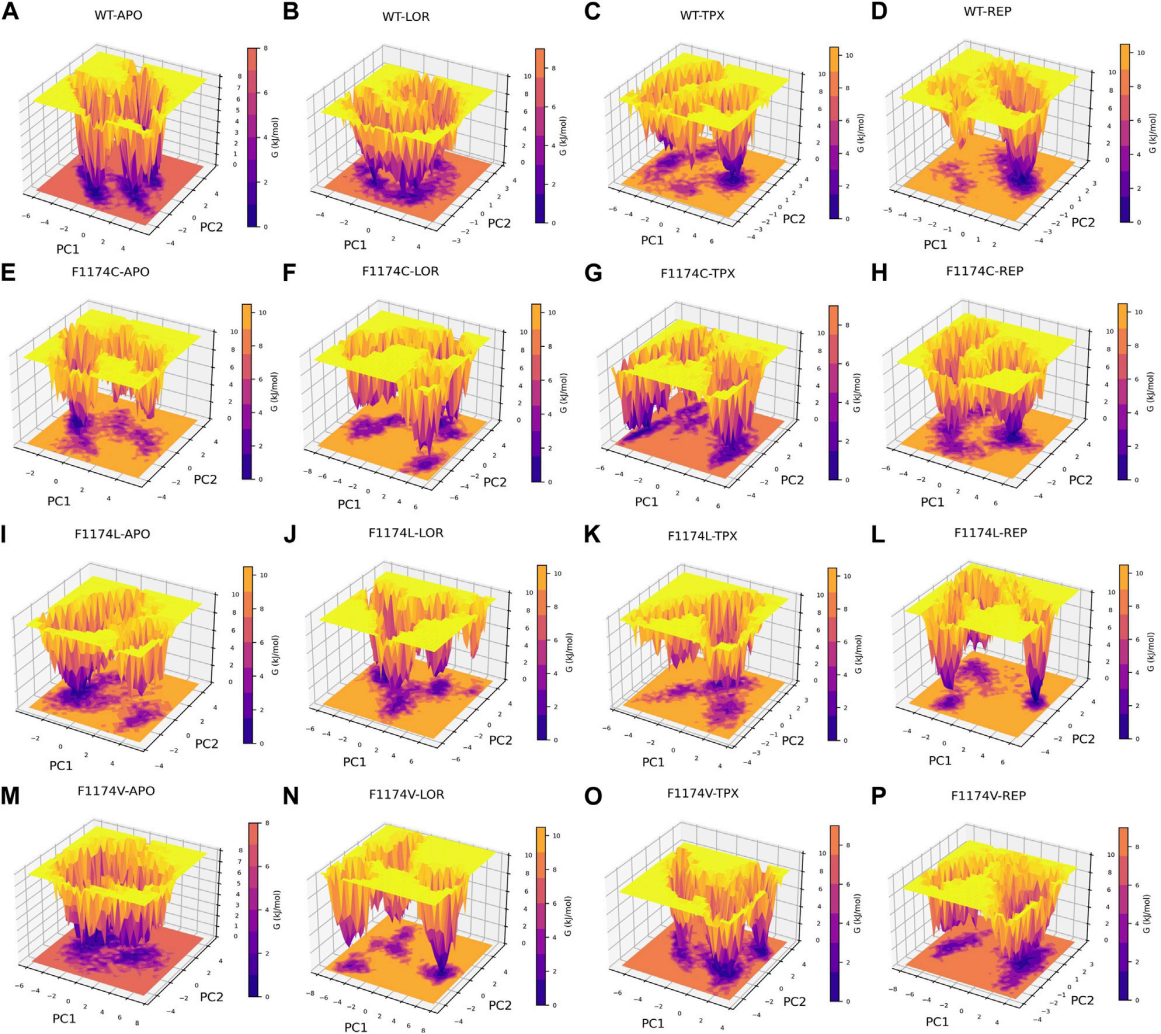
Gibbs free energy landscape, computed over a 200 ns MDS for ALK, is presented for the following conditions: **(A)** WT–APO, **(B)** WT–LOR, **(C)** WT–TPX, **(D)** WT–REP **(E)** F1174C–APO, **(F)** F1174C–LOR, **(G)** F1174C–TPX, **(H)** F1174C–REP **(I)** F1174L–APO, **(J)** F1174L–LOR, **(K)** F1174L–TPX, **(L)** F1174L–REP **(M)** F1174V–APO, **(N)** F1174V–LOR, **(O)** F1174V–TPX, and **(P)** F1174V–REP. The color gradient, ranging from purple (minimum energy) to yellow (maximum energy), represents the Gibbs free energies (in kJ/mol) of the various conformational states.

Gibbs free energy surface was calculated using the first two PCs (PC1 and PC2). The FES determined for ALK WT and F1174C/L/V APOs and complexes individually and each system is depicted in Figure 9. The color bar represents the Gibbs free energies in kJ/mol for the structural states, ranging from the lowest energy state in lavender to the highest in orange. Compared to WT, the F1174C/L/V mutations showed a significant difference in the Gibbs free energy (Figure 9). The Gibbs free energy of WT (7.87 kJ/mol) is very low compared with the Gibbs free energy of F1174C (9.67 kJ/mol) and F1174L (9.17 kJ/mol), whereas there are no significant changes in the Gibbs free energy of F1174V (7.87 kJ/mol). A deeper lavender color indicates the larger area of various conformational states with lower energy minima and represents a stable cluster. In WT-APO, three similar stable clusters formed, in which we noticed a stable cluster in F1174V–APO, various in F1174C/L, and several lower energy minima indicating unstable structures. The Gibbs free energy for the WT–LOR (8.55 kJ/mol) complex is low compared with WT–TPX (9.59 kJ/mol) and WT–REP (9.74 kJ/mol). The Gibbs free energy for the F1174C–TPX (8.66 kJ/mol) complex is low compared with F1174C–LOR (9.51 kJ/mol) and F1174C–REP (9.17 kJ/mol). The Gibbs free energy for the F1174L–LOR (9.51 kJ/mol) and F1174L–TPX (9.74 kJ/mol) complexes is low compared with F1174L–REP (10.4 kJ/mol). The Gibbs free energy for the F1174V–TPX (8.30 kJ/mol) and F1174V–REP (8.77 kJ/mol) complexes is low compared with F1174V–LOR (10.5 kJ/mol). The lower energy minima, enriched and spread across a wide space, have been observed for WT–LOR, F1174C–TPX, F1174L–LOR, F1174L–TPX, F1174V–TPX, and F1174V–REP complexes represented in lavender color compared to other complexes. Thus, it states that these complexes occupy a wider lavender region and signify stable structures.

#### 3.2.7 Protein stability and flexibility dynamics analysis

**FIGURE 10:**
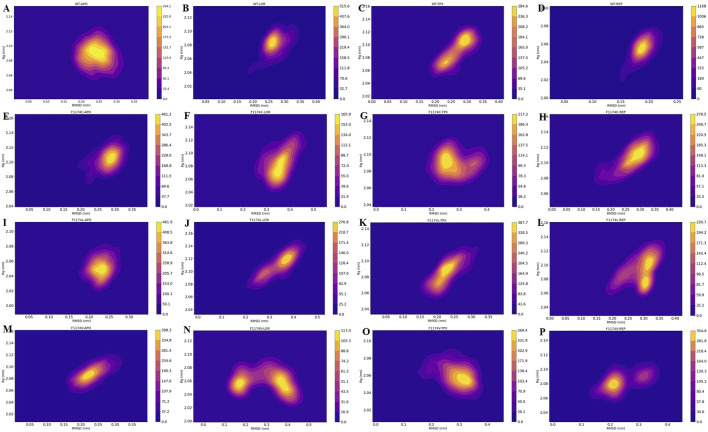
RMSD vs. Rg obtained over the 200 ns MDS for ALK **(A)** WT–APO, **(B)** WT–LOR, **(C)** WT–TPX, **(D)** WT–REP **(E)** F1174C–APO, **(F)** F1174C–LOR, **(G)** F1174C–TPX, **(H)** F1174C–REP **(I)** F1174L–APO, **(J)** F1174L–LOR, **(K)** F1174L–TPX, **(L)** F1174L–REP **(M)** F1174V–APO, **(N)** F1174V–LOR, **(O)** F1174V–TPX, and **(P)** F1174V–REP. The *x*-axis shows the RMSD in nm, while the *y*-axis shows the Rg in nm. The color bar represents the stability and flexibility of protein conformational states with the most (yellow) and least (orange–purple) conformations.

The probability density function (PDF) of RMSD and Rg were computed for ALK WT and F1174C/L/V APOs and complexes to assess the most dense conformation occupied during MDS (Figure 10). The estimated PDF is based on the kernel density estimate (KDE), which uses statistical tools to identify the most frequent molecular conformations. The PDF’s deepest and most concentrated areas signify the protein’s flexibility and stability. According to the PDF analysis, the most occupied conformation of WT–APO, F1174C–APO, F1174L–APO, and F1174V–APO can be identified at RMSD 0.24 nm and Rg 2.07 nm, RMSD 0.29 nm and Rg 2.11 nm, RMSD 0.24 nm and Rg 2.05 nm, and RMSD 0.22 nm and Rg 2.08 nm, respectively. The most occupied conformations for WT–LOR, WT–TPX, WT–REP, F1174C–LOR, F1174C–TPX, F1174C–REP, F1174L–LOR, F1174L–TPX, F1174L–REP, F1174V–LOR, F1174V–TPX, and F1174V–REP were identified at RMSD 0.26 nm and Rg 2.08 nm, RMSD 0.29 nm and Rg 2.11 nm, RMSD 0.18 nm and Rg 2.05 nm, RMSD 0.35 nm and Rg 2.07 nm, RMSD 0.24 nm and Rg 2.07 nm, RMSD 0.29 nm and Rg 2.11 nm, RMSD 0.38 nm and Rg 2.11 nm, RMSD 0.28 nm and Rg 2.09 nm, RMSD 0.30 nm and Rg 2.07 nm, RMSD 0.35 nm and Rg 2.06 nm, RMSD 0.33 nm and Rg 2.05 nm, and RMSD 0.23 nm and Rg 2.08 nm (Figure 10). The yellow color represents more dynamically stable conformations, and the orange–purple gradient color represents less dynamically stable conformations.

#### 3.2.8 Dynamics of secondary structure analysis

**FIGURE 11:**
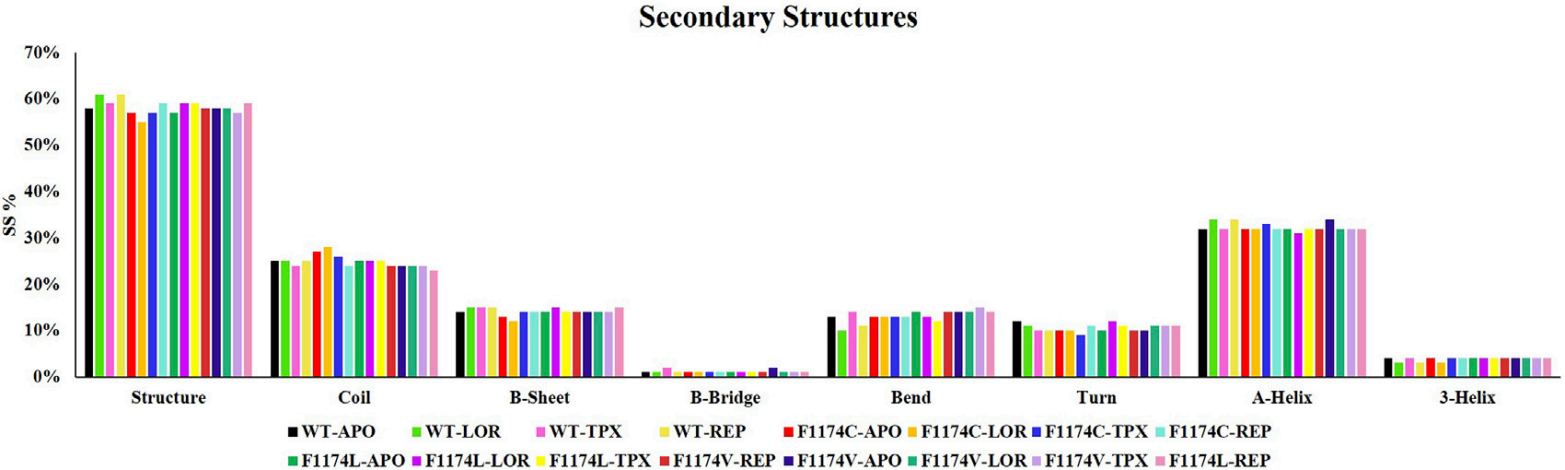
Percentage of residues involved in the secondary structure content of ALK WT and F1174C/L/V mutations with and without LOR, TPX, and REP inhibitors.

Secondary structure analysis was used to look at changes in the secondary structures for ALK WT and F1174C/L/V APOs and complexes using the dictionary of secondary structure of protein (DSSP) analysis. This analysis allowed us to understand the protein’s conformational activity and folding mechanisms. The time evolution of the secondary structures for APOs and complexes of ALK WT and F1174C/L/V is shown individually in Supplementary Figure S2. The secondary structural elements (structure (A-helix + B-sheet + B-bridge + turn), coil, B-sheet, B-bridge, bend, turn, A-helix, and 3-helix) were split by the protein into individual residues at each time step, and the mean residues establishing the secondary structure were seen as a function of time. The investigation demonstrates that the APO structural components of ALK are almost constant and equilibrated throughout the simulation. A slight increase in the ALK secondary structure element is visible in complexes with inhibitors. This increase in secondary structure content is mostly the result of the transformation of coils into helices. The secondary structure content did not significantly alter after binding, indicating the high stability of all complexes. The average secondary structural elements for all the protein structures are depicted in a bar chart (Figure 11).

#### 3.2.9 Time-correlated dynamics cross-correlation map of backbone atom motions

**FIGURE 12:**
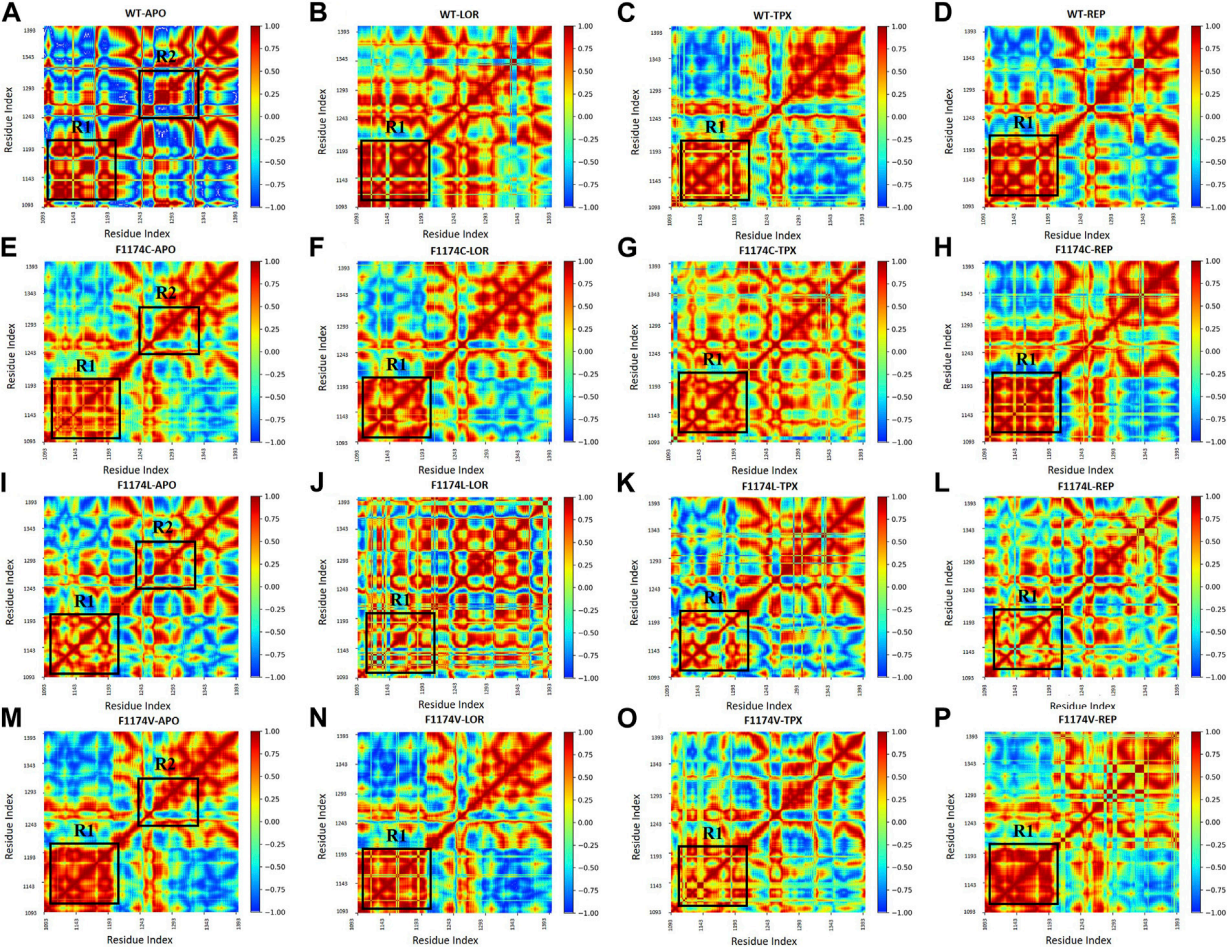
DCCM calculated using the coordinates of the backbone atoms from ALK **(A)** WT–APO, **(B)** WT–LOR, **(C)** WT–TPX, **(D)** WT–REP **(E)** F1174C–APO, **(F)** F1174C–LOR, **(G)** F1174C–TPX, **(H)** F1174C–REP **(I)** F1174L–APO, **(J)** F1174L–LOR, **(K)** F1174L–TPX, **(L)** F1174L–REP **(M)** F1174V–APO, **(N)** F1174V–LOR, **(O)** F1174V–TPX, and **(P)** F1174V–REP. The color bar represents the co-related motions of the residues with positive (red) and negative (blue) co-related movements.

The DCCM is a two-dimensional matrix that displays correlation in the residue movements for the timeline of MDS. The red-colored sections exhibit a positive correlation, the blue-colored sections exhibit a negative correlation in protein residue movement, and the yellow portions show no association. The strongly correlated matrix indicates the drug molecules’ strong contact with the protein binding site, resulting in coordinated movements in the overall protein structure. Compared to WT–APO, three different amino acid (C/L/V) substitutions at F1174 highly resemble the positive and negative correlated motions, mostly among the R1 and R2 regions (Figure 12). Figure 12 reveals that F1174C–REP, F1174L–REP, and F1174V–REP have the best and highest correlation among R1 regions compared to the LOR inhibitor. In contrast, TPX has lower correlations for F1174C/L/V, indicating that the F1174C/L/V mutation activity is reduced in REP-bounded complexes.

### 3.3 Binding free energy calculation using the MMPBSA analysis

**FIGURE 13:**
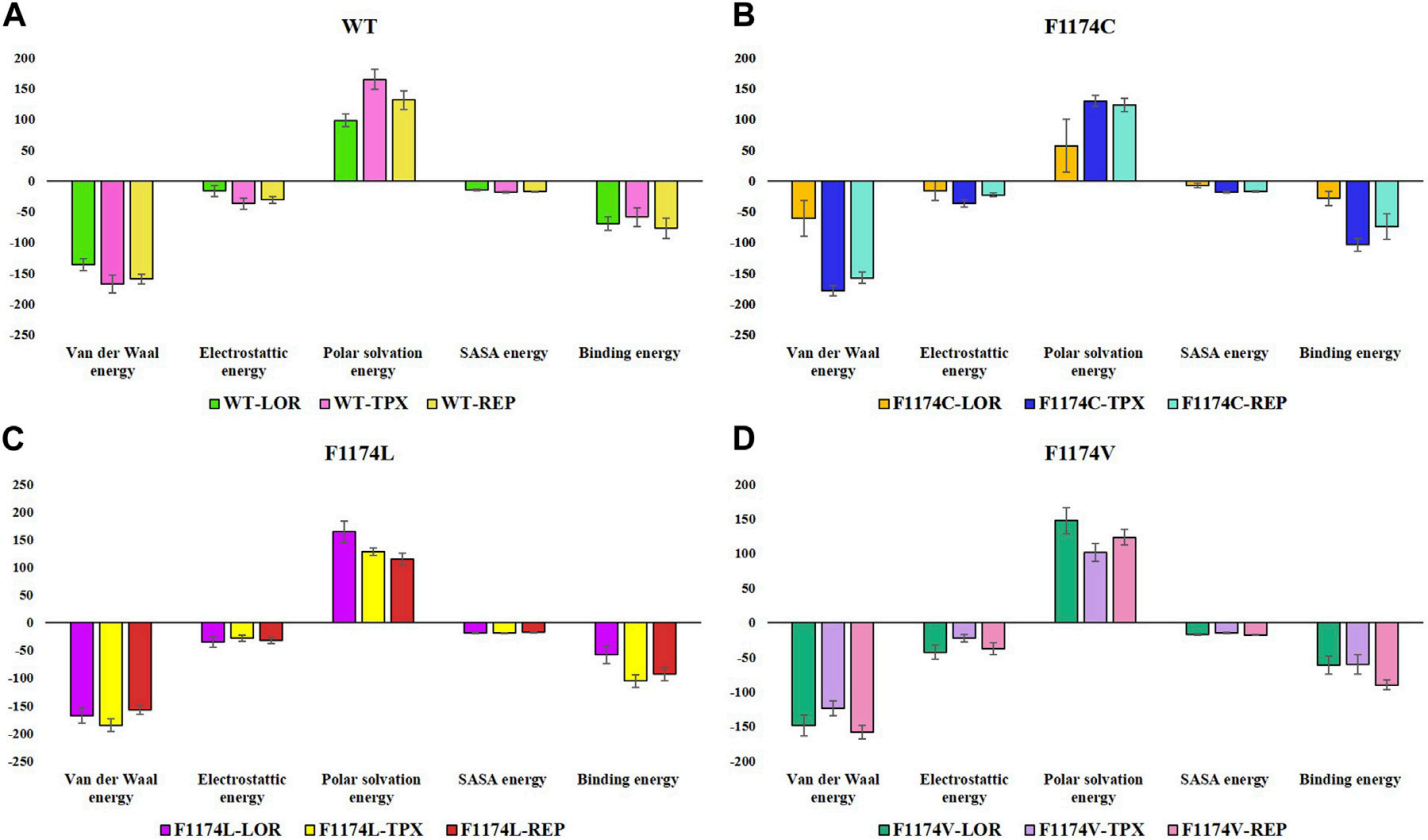
Overall binding energy contribution/decomposition plot of ALK WT and F1174C/L/V mutations complexed with LOR, TPX, and REP inhibitors using the MMPBSA calculation. **(A)** The overall binding energy contribution comparison of WT–LOR, WT–TPX, and WT–REP. **(B)** The overall binding energy contribution comparison of F1174C–LOR, F1174C–TPX, and F1174C–REP. **(C)** The overall binding energy contribution comparison of F1174L–LOR, F1174L–TPX, and F1174L–REP. **(D)** The overall binding energy contribution comparison of F1174V–LOR, F1174V–TPX, and F1174V–REP.

**FIGURE 14:**
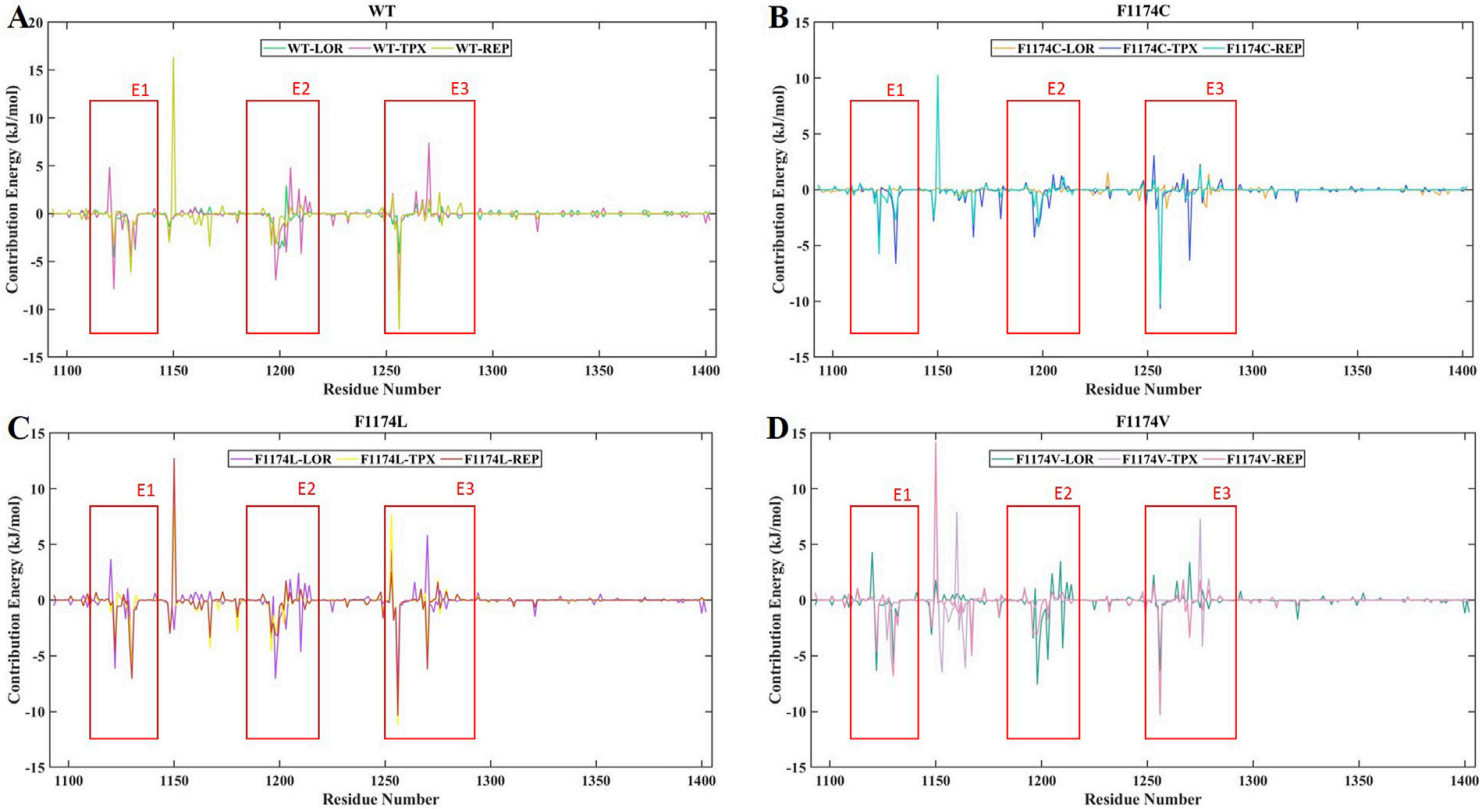
MMPBSA binding energy contribution per residue of ALK WT and F1174C/L/V mutations complexed with inhibitors LOR, TPX, and REP. **(A)** Residue-wise binding energy contribution comparison of WT–LOR, WT–TPX, and WT–REP. **(B)** Residue-wise binding energy contribution comparison of F1174C–LOR, F1174C–TPX, and F1174C–REP. **(C)** Residue-wise binding energy contribution comparison of F1174L–LOR, F1174L–TPX, and F1174L–REP. **(D)** Residue-wise binding energy contribution comparison of F1174V–LOR, F1174V–TPX, and F1174V–REP.

All simulated complexes’ binding affinities were evaluated by estimating the binding free energies of each one using the MMPBSA analysis. The final 50 ns of the MDS trajectories were used to estimate the binding free energies. The binding free energies of each system were associated with their van der Waals, electrostatic, polar solvation, and SASA energies, which were determined and shown in Table 3. The estimated binding energy for the WT–LOR, WT–TPX, WT–REP, F1174C–LOR, F1174C–TPX, F1174C–REP, F1174L–LOR, F1174L–TPX, and F1174L–REP was −69.516 +/11.094 kJ/mol, −59.082 ± 15.181 kJ/mol, −77.129 ± 16.377 kJ/mol, −28.410 ± 11.471 kJ/mol, −104.052 ± 10.196 kJ/mol, −74.561 ± 20.828 kJ/mol, −57.904 ± 16.262 kJ/mol, −105.019 ± 11.487 kJ/mol, −92.811 ± 12.691 kJ/mol, −61.370 ± 13.177 kJ/mol, −60.302 ± 14.341 kJ/mol, and −89.999 ± 6.851 kJ/mol (Figure 13; Table 3). The WT–REP complex exhibited a higher binding energy in WT than WT–LOR and WT–TPX. In F1174C and F1174L complexes, TPX exhibited a higher binding energy compared to others. Meanwhile, in F1174V, REP exhibited a higher binding energy. The residual binding energy calculations of the simulated complexes were carried out to pinpoint the amino acid residues essential for inhibitor binding (Figure 14). All the ligands were shown to have a substantial role in the interactions with the protein’s amino acid residues, suggesting the possibility of ALK inhibitors. Positions 1120 to 1145 (E1), 1190 to 1215 (E2), and 1250 to 1290 (E3) of the amino acid residues made a greater contribution to interactions in all the complexes (Figure 14).

**TABLE 3:** Summary of binding energy analysis using the MMPBSA calculation for ALK WT and F1174C/L/V mutations complexed with LOR, TPX, and REP inhibitors.

	van der Waals energy (kJ/mol)	Electrostatic energy (kJ/mol)	Polar solvation energy (kJ/mol)	SASA energy (kJ/mol)	Binding energy (kJ/mol)
WT–LOR	−136.043 ± 9.521	−16.699 ± 8.862	98.160 ± 10.395	−14.934 ± 0.633	−69.516 ± 11.094
WT–TPX	−167.886 ± 14.242	−37.084 ± 9.332	164.744 ± 16.186	−18.857 ± 1.159	−59.082 ± 15.181
WT–REP	−159.781 ± 8.042	−30.971 ± 5.670	131.259 ± 15.301	−17.636 ± 0.407	−77.129 ± 16.377
F1174C–LOR	−61.441 ± 29.175	−16.087 ± 15.731	57.061 ± 43.167	−7.943 ± 3.476	−28.410 ± 11.471
F1174C–TPX	−178.529 ± 8.633	−36.391 ± 6.046	129.590 ± 9.029	−18.722 ± 0.777	−104.052 ± 10.196
F1174C–REP	−157.509 ± 8.856	−23.112 ± 2.974	123.376 ± 10.945	−17.315 ± 0.928	−74.561 ± 20.828
F1174L–LOR	−168.098 ± 14.221	−34.961 ± 9.570	163.989 ± 19.184	−18.834 ± 1.133	−57.904 ± 16.262
F1174L–TPX	−185.303 ± 11.613	−27.979 ± 5.616	127.572 ± 6.873	−19.308 ± 0.806	−105.019 ± 11.487
F1174L–REP	−157.826 ± 8.290	−32.164 ± 5.764	115.047 ± 10.754	−17.868 ± 0.793	−92.811 ± 12.691
F1174V–LOR	−148.563 ± 15.273	−42.854 ± 10.168	147.372 ± 19.144	−17.325 ± 1.347	−61.370 ± 13.177
F1174V–TPX	−123.991 ± 10.873	−22.814 ± 5.500	101.193 ± 12.875	−14.691 ± 1.108	−60.302 ± 14.341
F1174V–REP	−158.121 ± 9.912	−37.508 ± 8.449	123.417 ± 10.965	−17.787 ± 0.628	−89.999 ± 6.851

## 4 Discussion

NSCLC is the predominant form of lung cancer, with *ALK* gene mutations accounting for approximately 5% of NSCLC cases. Patients with ALK-mutated NSCLC have better therapeutic options than conventional chemotherapy due to the development of first-generation crizotinib in targeted therapy (Cameron et al., 2019). However, most patients receiving crizotinib treatment develop resistance within a year. Crizotinib’s ability to penetrate the central nervous system (CNS) is limited, leading to CNS progression in 70% of patients with established brain metastases (Solomon et al., 2014). Following the approval of crizotinib, second-generation ALK inhibitors (alectinib, brigatinib, ceritinib, and ensartinib) have made significant advancements, showing greater effectiveness and improved responses within the brain for advanced ALK-positive NSCLC compared to crizotinib (Shaw et al., 2017b; Hida et al., 2017; Camidge et al., 2018; Horn et al., 2021). Later, lorlatinib was developed primarily to overcome resistance in mutants that do not respond well to first- and second-generation ALK TKIs. Lorlatinib showed significant efficacy in preclinical investigations using cell assays to block ALK phosphorylation and decrease cell growth across a range of ALK-resistant mutations. These mutations included frequent ones such as L1196M and G1269A (crizotinib resistance), as well as G1202R (first- and second-generation ALK TKI resistance) (Zou et al., 2015). The ALK mutation L1196M leads to varying levels of drug resistance against inhibitors. Free energy perturbation (FEP) and umbrella sampling simulations reveal that L1196M induces significant conformational changes in ALK, particularly affecting the flexibility of loops L1 and L2. Statistical analysis highlights a decrease in hydrophobic interactions with residue L1256 as a major contributor to drug resistance, providing insights for designing potent inhibitors against L1196M-induced resistance in ALK (Chen et al., 2017; Chen et al., 2020) used MR-GaMD (multiple replica Gaussian accelerated molecular dynamics), MM-GBSA, and free energy landscapes to investigate the impact of mutations (L1198F, L1198F/C1156Y, and C1156Y) on crizotinib binding to ALK. Findings reveal that L1198F and L1198F/C1156Y enhance binding, while C1156Y induces drug resistance. The analysis highlights specific residues as potential targets for anti-drug-resistance designs (Chen et al., 2020). An early study revealed that F1174L brings the αC-helix and αAL-helix closer together, distorting the distal part of the A-loop, whereas WT-ALK and R1275 play a dual role in maintaining the inactive αAL-helix–αC-helix interaction and securing αAL-helix conformation via the D1276–R1275 interaction. Mutating R1275 to glutamine causes the αC-helix to become active and distorts the whole A-loop. F1174L and R1275Q mutants both rearrange the A-loop, exposing the P+1 pocket and reactivating kinase activity (Jiang et al., 2018).

The spectrum of ALK resistance mutations varies, with 20% of crizotinib patients developing ALK-resistant mutations and 56% of individuals developing resistance while on second-generation TKIs. Among individuals who previously received treatment with first-/second-generation AK inhibitors, the most frequently observed ALK mutations were G1202R (40%), F1174X (20%), I1171X (13%), and G1269A (13%), all of which have shown sensitivity to lorlatinib (Yun and Bazhenova, 2022). As lorlatinib remains the sole third-generation ALK TKI available for advanced ALK-positive NSCLC, there is a crucial requirement for further investigations on determining novel approaches to combat resistance that may arise during treatment progression. A promising approach includes designing more compact and smaller macrocyclic ALK inhibitors to develop effective ALK-targeted therapy and to overcome the existing drug resistance. The small, compact macrocyclic drug TPX-0131, undergoing an IND-enabling investigation, has also been demonstrated to have extremely powerful actions against most compound mutations that confer resistance to lorlatinib (Murray et al., 2021a). Another drug called repotrectinib, an ALK TKI drug, is being explored in phase I clinical studies to treat patients who are resistant to ALK TKIs because of their excellent effectiveness in overcoming ALK drug-resistant mutations, including L1196M and G1202R mutations (Drilon et al., 2018). The next-generation (fourth-generation) ALK TKIs were expected to be macrocyclic inhibitors (Song et al., 2021). New-generation TKIs have improved CNS penetration across the blood–brain barrier to achieve greater intracranial response rates and prevent brain metastases. There is still a need for a side-by-side comparison of the existing third-generation lorlatinib and fourth-generation TKIs. This current study explored the effect of F1174C/L/V missense mutations on the ALK structure and compared the binding affinity of lorlatinib and fourth-generation TPX-0131 and repotrectinib TKIs via a computational approach.

A molecular docking study was undertaken using lorlatinib, TPX-0131, and repotrectinib TKIs for examining the binding impact on F1174C/L/V mutations and their molecular functions of the ALK protein. Different amino acid substitutions drastically altered the binding pocket from the molecular docking analysis of ALK WT and F1174C/L/V mutations with TKIs such as lorlatinib, TPX-0131, and repotrectinib. Compared to WT-ALK, F1174C/L/V mutations with inhibitors showed significant binding energies and H-bond interactions. F1174C–REP, F1174C–LOR, F1174L–TPX, F1174V–LOR, and F1174V–TPX showed the least Ki values and significant binding energy and H-bond interactions compared to other complexes (Table 1; Figure 1).

RMSD graphs reveal divergence in convergence among the various complexes. The RMSD curve reliably flattened after 60 ns in all simulations, indicating that all structures reached equilibrium. This RMSD analysis suggests we can proceed with further analysis (Figure 2). According to the RMSF analysis (Figure 3), the overall flexibility of F1174C/L/V mutations does not show significant changes compared to ALK WT. In contrast, rigidity was observed in the F1174L/V (A1 region), F1174C/V (A2 region), and F1174C/L/V (A3 region) mutations. Furthermore, an inspection of the Cα atoms’ RMSF values revealed a consistent and dynamically stable structure. In comparing the drug’s inhibitory effects of RMSF analysis in the ALK WT context, the TPX inhibitor-bound complex showed greater flexibility. In contrast, the LOR and REP inhibitor-bound complexes exhibited rigidity. In the F1174C mutation, all inhibitor-bound complexes demonstrated rigidity. For the F1174L mutation, the TPX inhibitor-bound complex remained flexible, while the LOR and REP complexes remained rigid. In the case of the F1174V mutation, the TPX inhibitor-bound complex displayed flexibility, while the LOR and REP complexes maintained their rigidity (Figure 4).

According to the Rg analysis (Figure 5), the WT–REP complex showed a slightly flexible structure compared to the WT–APO complex, while WT–LOR and WT–TPX complexes remained relatively unchanged. In the case of the F1174C mutation, there were no notable differences in the complexes compared to the F1174C–APO structure. These results suggest no major changes in the compactness of the F1174C structure after being bound with an inhibitor. In the F1174L mutation, all the inhibitor-bound complexes exhibited a more compact structure than F1174L–APO. In F1174V complexes, LOR/TPX/REP inhibitor-bound F1174V complexes were slightly stretched (flexible) compared to those in F1174V–APO.

The F1174C mutation led to more exposure to solvent surface area, while the F1174L and F1174V mutations resulted in less exposure. The mutant inhibitor-bound complexes generally had a significantly higher mean SASA than the WT complexes. This analysis suggests that in mutants, more surface area is available for interaction with the solvent, except in F1174L–REP and F1174V–REP complexes (Figure 6). The F1174C/L/V mutations did not induce notable alterations in the count of intramolecular hydrogen bonds (Supplementary Figure S1). However, the WT inhibitor-bound complexes observed increased intramolecular H-bonds compared to the WT–APO complex, indicating that the inhibitor binding made the structure more stable. The F1174C–LOR complex exhibited minor disruption in intramolecular H-bonds compared to other inhibitor-bound complexes of F1174C. In F1174L, all the inhibitor bindings enhanced the structure’s stability. Similarly, TPX and REP binding to F1174C resulted in greater stability than LOR. The secondary structure comparison remained largely unchanged following the inhibitor binding, demonstrating the strong stability of all complexes (Figure 11).

Using PCA and FES (Figures 8, 9), we identified the stable inhibitor-bound structures, such as WT–TPX, WT–REP, F1174C–TPX, F1174C–REP, F1174L–LOR, and F1174V–LOR, with lower energy minima, which are denoted by a broad lavender region. Furthermore, we calculated the PDF for RMSD and Rg in ALK WT and F1174C/L/V APOs and complexes. We identified the most densely populated conformation during MDS in all the structures that occurred within an RMSD of ≤0.35 and Rg of ≤2.11 are considered stable complex structures (Figure 10).

ALK’s three hotspot residues, F1174, R1275, and F1245, are positioned around the αC-helix. Mutations at these sites change ALK into an active kinase. The increased kinase activity can be elucidated by considering the interconnected “communities” within the kinase domain. Notably, F1174 and R1275 are central amino acids within a hydrophobic core between the αC-helix and the catalytic loop, keeping the kinase domain in an auto-inhibited conformation. Mutations at F1174 and R1275 will destabilize ALK’s autoinhibitory interactions and facilitate kinase activation (Hallberg and Palmer, 2016). Residues close to the αC-helix are affected by F1174C/L/V mutations. A computational study revealed the allosteric effect of ALK F1174C mutation to ceritinib in lung cancer, and this is due to mutation affecting the interaction between ceritinib and the P-loop by changing the conformational dynamics of the P-loop and causing it to shift upward from the ATP-binding site (Ni et al., 2016).

Intermolecular H-bonds exhibit strong persistence in the following complexes: WT–TPX, WT–REP, F1174C–TPX, F1174C–REP, F1174L–LOR, and F1174V–LOR (Figure 7). The greater the number of H-bonds formed and the longer the life of H-bonds, the higher the binding affinity. We further delved deeper into the phenomenon of H-bond formation to pinpoint which specific residues within the binding site were pivotal in facilitating these interactions (Torshin et al., 2002). We analyzed the H-bond occupancy percentage on vmd by applying specific criteria: a donor–acceptor distance of 3 and an angle cut-off of 20. We obtained detailed information on the unique H-bonds (Supplementary Figure S3). The outcomes of the H-bond occupancy analysis are depicted in Supplementary Figure S3. WT–LOR formed nine unique H-bond interactions, notably with the residue Asp1203 (12.65%). WT–TPX formed five unique H-bond interactions, notably with the residues Glu1197 (24.24%) and Met1199 (38.83%). WT–TPX displayed the highest occupancy percentage, suggesting a substantial contribution to the system’s stability. F1174C–LOR formed 32 unique H-bond interactions, notably with the residue Asn1335 (6.95%). WT–REP formed two unique H-bonds, notably with the residue Met1199 (8.25%). F1174C–TPX formed three unique H-bond interactions, notably with the residue Met1199 (11.34%). F1174C-REP formed seven unique H-bonds, notably with the residue Met1199 (12.04%). F1174L–LOR formed five unique H-bond interactions, notably with the residues Glu1197 (41.18%) and Met1199 (32.78%). F1174L–TPX formed three unique H-bond interactions, notably with the residue Met1199 (8.60%). F1174L–REP formed three unique H-bonds, notably with the residue Met1199 (10.64%). F1174V–LOR formed four unique H-bond interactions, notably with the residues Glu1197 (35.43%), Met1199 (37.73%), and Asp1270 (7.25%). F1174V–TPX formed seven unique H-bond interactions, notably with the residue Glu1129 (1.35%). F1174V–REP formed four unique H-bonds, notably with the residue Met1199 (11.29%). In the case of TPX, it was observed that WT and F1174C mutations exhibited the highest H-bond occupancy levels than other inhibitors. LOR showed greater occupancy levels in ALK WT and F1174C/L/V mutations. However, TPX and REP showed greater H-bond occupancy compared to LOR in F1174C alone.

Earlier studies observed that mutations occurring at the active site led to steric interference, causing a change in ligand binding affinity and resulting in resistance to ALK inhibitors. Furthermore, for numerous drug-resistant ALK mutations, enhanced ATP-binding affinity has also been reported (Li et al., 2018). Similarly, the existence of a closed (occluded) drug binding site or a decrease in lorlatinib binding energy were both considered as potential causes of resistance in the computational kinetic model of lorlatinib competitive binding to ALK TKD in the presence of ALK (Berko et al., 2023). Lorlatinib exhibits notable clinical benefits, particularly in advanced ALK-positive NSCLC with intensive previous treatment (Baldacci et al., 1990). However, it has been discovered that patients with ALK fusion NSCLC who develop compound ALK TKD mutations while receiving treatment exhibit decreased sensitivity to lorlatinib and clinical development (Shiba-Ishii et al., 2022). ALK mutations were found in 76% of plasma specimens in patients, and patients whose condition worsened while on lorlatinib treatment had the following mutations: 38% of L1196M, 28% of G1202R, 24% of D1203N, 14% of F1174C/L, and 14% of I1171X. These include single and compound mutations, with compound mutations occurring between 35% and 48% of patients receiving treatment (Yoda et al., 2018; Dagogo-Jack et al., 2019).

According to the MMPBSA and per residue binding energy calculations (Figures 13, 14), the WT–REP complex had greater binding energy compared to the other WT complexes (WT–LOR and WT–TPX). TPX showed the highest binding energy with F1174C and F1174L complexes. On the other hand, REP has the highest binding energy in the F1174V complex. This outcome supports the research’s conclusions from the earlier study.

ALK fusion proteins are inhibited by TPX-0131, a small macrocyclic molecule that is made to fit inside the ATP-binding site. TPX-0131 inhibitor exhibited greater efficacy than approved ALK inhibitors compared to wild-type ALK and various ALK resistance mutations, including L1196M, G1202R, and compound mutations. In biochemical assessments, TPX-0131 demonstrated potent inhibition of wild-type and 26 ALK mutants, encompassing single and compound mutations. Notably, TPX-0131 effectively halted tumor growth in ALK G1202R and ALK compound mutations, whereas lorlatinib did not exhibit the same level of suppression. (Murray et al., 2021b). Repotrectinib suppresses the phosphorylation of ALK mutants that are constitutively active. Repotrectinib, with a dosage of 12–26 nm, showed sensitivity in ALK WT, G1128A, I1171N, F1174L, R1192P, F1245C, and Y1278S, whereas R1275Q and EML4-ALK secondary mutations resembling G1269A required a larger dosage to suppress Y1604 phosphorylation in neuroblastoma cells. In comparison, repotrectinib suppressed constitutively active ALK more effectively than crizotinib (Drilon et al., 2018). Our findings show that the fourth-generation binding affinity for F1174C/L/V mutations suggests that these drugs may be able to overcome lorlatinib resistance.

## 5 Conclusion

The current study provided insights into the binding mechanism of fourth-generation TKIs (TPX-0131 and repotrectinib) for ALK F1174C/L/V mutations over the third-generation TKI lorlatinib. TPX-0131 and repotrectinib showed more promising MDS results than lorlatinib, accompanied by stable intermolecular H-bond interaction and binding energy during the simulation. Together, these *in silico* findings support the implementation of fourth-generation TKIs for ALK-positive NSCLC treatment.

## Data Availability

The original contributions presented in the study are included in the article/Supplementary Material; further inquiries can be directed to the corresponding author.

## Supplementary material

SUPPLEMENTARY FIGURE S1 Analysis of the number of intramolecular H-bond formations in ALK WT and F1174C/L/V mutations with and without inhibitors. **(A)** The comparison of the number of intramolecular H-bond formations in WT–APO, WT–LOR, WT–TPX, and WT–REP. **(B)** The comparison of the number of intramolecular H-bond formations in F1174C–APO, F1174C–LOR, F1174C–TPX, and F1174C–REP. **(C)** The comparison of the number of intramolecular H-bond formations in F1174L–APO, F1174L–LOR, F1174L–TPX, and F1174L–REP. **(D)** The comparison of the number of intramolecular H-bond formations in F1174V–APO, F1174V–LOR, F1174V–TPX, and F1174V–REP. The *x*-axis represents the time in nanoseconds (ns), while the *y*-axis indicates the number of intermolecular H-bonds formed.

SUPPLEMENTARY FIGURE S2 The time evolution of the secondary structure content of ALK WT and F1174C/L/V mutations with and without inhibitors (LOR, TPX, and REP) over the 200 ns MDS.

SUPPLEMENTARY FIGURE S3 Hydrogen bond occupancy percentage for protein–ligand complexes. **(A)** WT–LOR, **(B)** WT–TPX, **(C)** WT–REP **(D)** F1174C–LOR, **(E)** F1174C–TPX, **(F)** F1174C–REP **(G)** F1174L–LOR, **(H)** F1174L–TPX, **(I)** F1174L–REP **(J)** F1174V–LOR, **(K)** F1174V–TPX, and **(L)** F1174V–REP.
