# Supplementary material for: A computational examination of the therapeutic advantages of fourth-generation ALK inhibitors TPX-0131 and repotrectinib over third-generation lorlatinib for NSCLC with ALK F1174C/L/V mutations
Source: Front Mol Biosci. 2024 Jan 11;10:1306046. doi: 10.3389/fmolb.2023.1306046 (PMC10808363; doi:10.3389/fmolb.2023.1306046)
Supplement: Supplementary file 1 [file Image1.pdf]

Supporting Information for:

A Computational Examination of the Therapeutic Advantages of Fourth-Generation ALK Inhibitors TPX-0131 and Repotrectinib over Third-Generation Lorlatinib for NSCLC with ALK F1174C/L/V Mutations

Ambritha Balasundaram and George Priya C Doss *

Laboratory of Integrative Genomics, Department of Integrative Biology, School of BioSciences and Technology, Vellore Institute of Technology, Vellore, Tamil Nadu 632014, India; ambritha.b2020@vitstudent.ac.in (AB); ORCiD iD: https://orcid.org/0000-0003-2856-4687; georgepriyadoss@vit.ac.in (CGPD); ORCiD iD: https://orcid.org/0000-0002-5971-8290

*Correspondence: George Priya C Doss, Laboratory of Integrative Genomics, Department of Integrative Biology, School of Biosciences and Technology, Vellore Institute of Technology, Vellore, India; georgepriyadoss@vit.ac.in

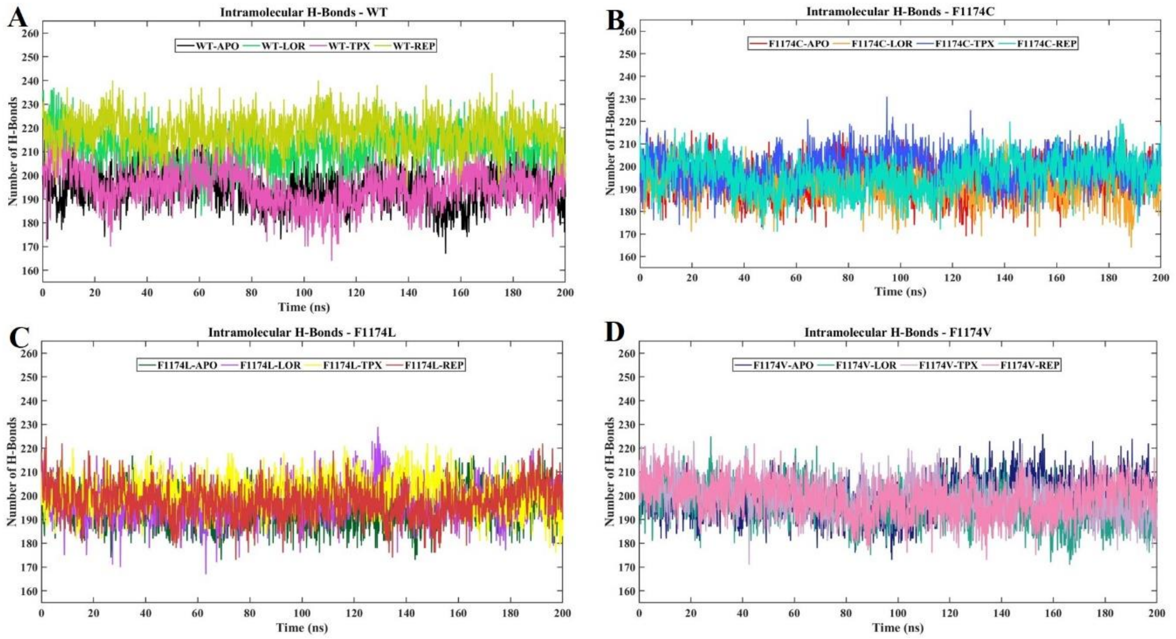

Supplementary Figure 1: Analysis of the number of **i**ntramolecular H-bond formations in ALK WT and F1174C/L/V mutations with and without inhibitors. **(A)** The comparison of the number of **i**ntramolecular H-bond formations in WT-APO, WT-LOR, WT-TPX, and WT-REP. **(B)** The comparison of the number of **i**ntramolecular H-bond formations in F1174C-APO, F1174C-LOR, F1174C-TPX, and F1174C-REP. **(C)** The comparison of the number of **i**ntramolecular H-bond formations in F1174L-APO, F1174L-LOR, F1174L-TPX, and F1174L-REP. **(D)** The comparison of the number of **i**ntramolecular H-bond formations in F1174V-APO, F1174V-LOR, F1174V-TPX, and F1174V-REP. The x-axis shows the time in ns, while the y-axis shows the number of intermolecular H-bonds formed.

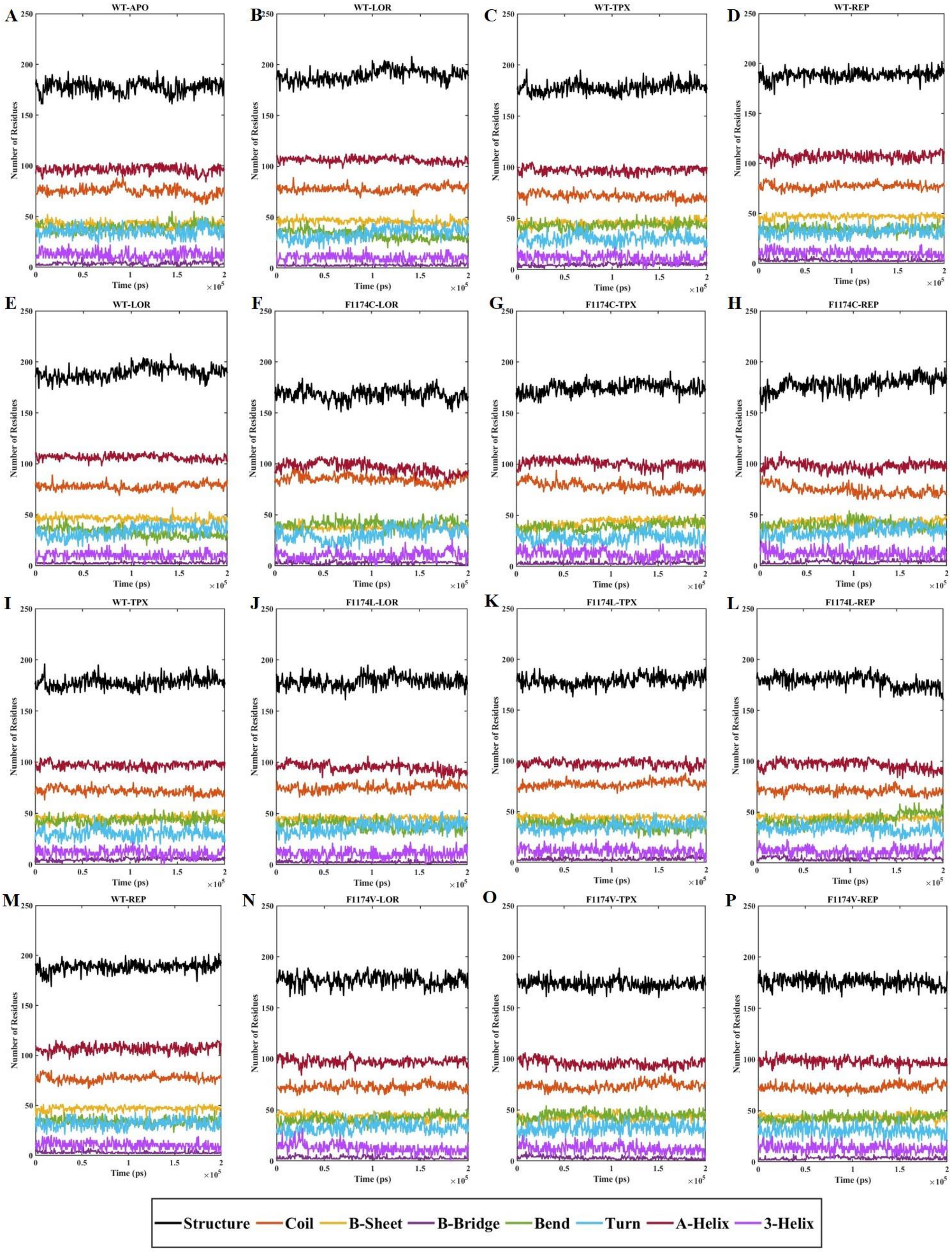

Supplementary Figure 2: The time evolution of the secondary structure content of ALK WT and F1174C/L/V mutations with and without inhibitors (LOR, TPX, and REP) over the 200 ns MDS.

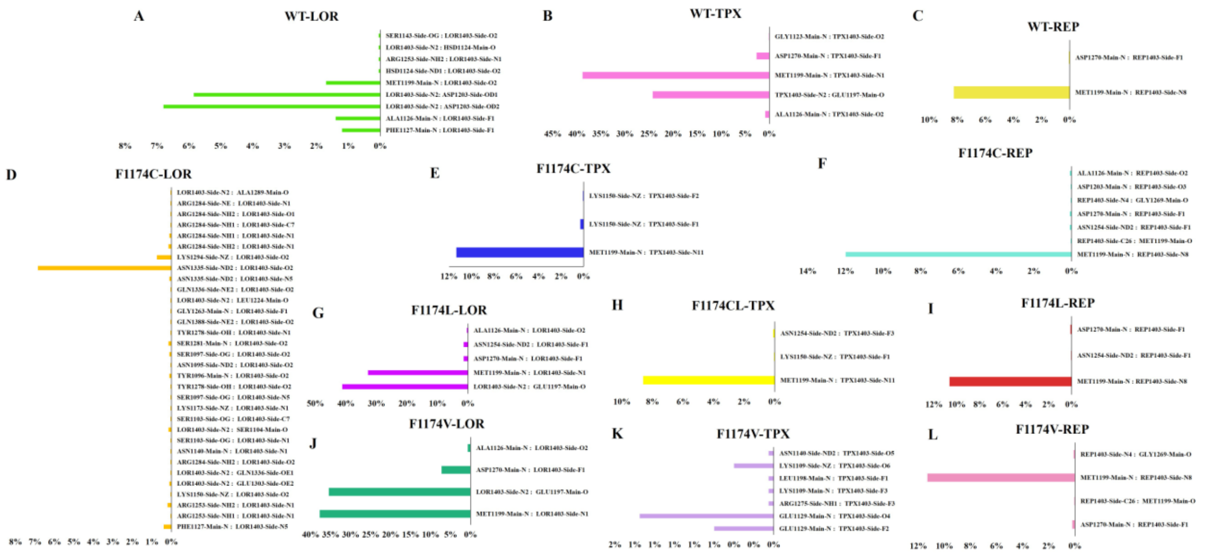

Supplementary Figure 3: Hydrogen bond occupancy percentage for the protein-ligand complexes. **(A)** WT-LOR, **(B)** WT-TPX, **(C)** WT-REP **(D)** F1174C-LOR, **(E)** F1174C-TPX, **(F)** F1174C-REP **(G)** F1174L-LOR, **(H)** F1174L-TPX, **(I)** F1174L-REP **(J)** F1174V-LOR, **(K)** F1174V-TPX, and **(L)** F1174V-REP.
